# Mapping the relationships among sleep, motor balance, and cognition in older adults: a systematic scoping review

**DOI:** 10.3389/fnhum.2025.1575155

**Published:** 2025-09-01

**Authors:** Yun Chan Shin, Shrinath Shah, Hailey Manoj Sejpal, Hongwu Wang

**Affiliations:** ^1^Department of Occupational Therapy, University of Florida, Gainesville, FL, United States; ^2^Department of Applied Physiology and Kinesiology, University of Florida, Gainesville, FL, United States

**Keywords:** cognition, sleep, older adults, systematic scoping review, motor balance

## Abstract

**Introduction:**

Sleep, motor balance, and cognitive function are critical for maintaining functional independence in older adults, yet their interrelationships remain poorly understood. This systematic scoping review maps the evidence on pairwise and triadic relationships among these domains in older adults.

**Methods:**

Following the JBI Manual for scoping reviews, we searched PubMed, Web of Science, CINAHL, and Embase for studies (January 1, 2004–March 1, 2024) involving older adults (≥60 years) that examined sleep, motor balance, and cognition. Covidence facilitated a two-phase screening, selecting studies assessing all three domains. Data on study design, participant characteristics, and outcome measures were extracted, with evidence levels assessed using NHMRC guidelines.

**Results:**

From 1,367 studies, 33 (7 experimental, 26 observational) involving 67,237 older adults were included. Sleep quality showed weak to moderate positive associations with motor balance (e.g., *r* = 0.1–0.3) and cognition, while motor balance confidence was positively linked to cognition. Only one study explored triadic interactions, revealing a significant gap. Effect sizes suggest limited clinical significance in some findings.

**Conclusion:**

Pairwise relationships among sleep, motor balance, and cognition are evident but weak. Longitudinal, multimodal intervention studies are needed to explore triadic interactions and inform integrated interventions.

## 1 Introduction

Sleep, motor balance, and cognitive function are well-known factors related to health, function, and mortality in older adults. Both insufficient or excessive sleep duration and insomnia symptoms are risk factors for mortality in older adults and for the incidence of adverse health conditions such as depression, cardiovascular disease, and type 2 diabetes (Antza et al., 2021; Cappuccio et al., 2010; Chaput et al., 2020; Nielson et al., 2023). Additionally, inadequate sleep duration is linked to insufficient physical activity engagement in older adults, and poor sleep quality is associated with impaired physical function and frailty (Arias-Fernández et al., 2021; Štefan et al., 2018). Motor balance deterioration is a major risk factor for falls in older adults, and falls are one of the leading causes of death in this population (Centers for Disease Control and Prevention, 2022; Tinetti et al., 1988). Furthermore, motor balance impairment leads to decreased mobility, resulting in reduced participation and promoting functional disability in older adults (Brown and Flood, 2013; Newman et al., 2006). Cognitive decline is a significant factor that can contribute to diminished quality of life, functional disability, and increased mortality in older adults (Macaulay et al., 2021; Villasán Rueda et al., 2021; Wei et al., 2020). Conversely, this implies that improving sleep quality, motor balance, and cognitive function may help promote healthy aging.

Efforts to prevent health-related adverse events by managing sleep, motor balance, and cognition have been ongoing. The early preventative intervention approach is well known for achieving better cognitive function or delaying cognitive decline in older adults (Hong et al., 2020; Li et al., 2023; Mowszowski et al., 2010). Research on interventions to manage sleep and motor balance is also active, and non-pharmacological interventions such as resistance exercise and Tai chi have shown significant effects on motor balance function and sleep (Efendi et al., 2023; Nguyen and Kruse, 2012). However, the limitation of existing research efforts is that sleep, motor balance, and cognition were often assessed separately, overlooking the complex interrelationships among sleep, motor balance, and cognition.

The relationship between sleep and motor balance has been established, suggesting that poor sleep duration and quality can impair motor balance, increasing the likelihood of adverse events like falls (Umemura et al., 2022). In addition, sleep duration shows an inverted U-shaped relationship with global cognition, memory, executive function, and orientation (Ma et al., 2020). Sleep duration significantly impacts cognitive function, with an optimal sleep duration of 6–7 h linked to improved cognitive function in older adults. Meanwhile, inadequate or excessive sleep has been correlated with cognitive decline. Meta-analyses have shown that better memory correlates with less restlessness at night, and better executive function and working memory correlate with shorter sleep onset latency (Qin et al., 2023). Lastly, poor standing motor balance is related to lower memory and overall cognition (Yan et al., 2022), and better motor balance is associated with superior executive function and processing speed (Divandari et al., 2023).

While relationships between sleep and motor balance, sleep and cognition, and motor balance and cognition were examined, the relationships among all three factors were poorly understood, which is critical for effective interventions and treatments to promote health and function in older adults. Therefore, this systematic scoping review aims to comprehensively synthesize the existing evidence on the relationships among sleep, motor balance, and cognition in older adults. The primary research question is: What are the identified relationships among sleep, motor balance, and cognition within the older adult population, based on current literature? By mapping the pattern of the relationships, this review will help identify research gaps and guide future research directions.

## 2 Materials and methods

We conducted a “Systematic scoping review” (Peters et al., 2015). This analysis brings together literature in disciplines with emerging evidence to address questions beyond those related to the effectiveness or experience of an intervention (Peters et al., 2020). It examines a broader area to identify gaps in the research knowledge base, clarify key concepts, and report on the types of evidence that address and inform practice in the field (Peters et al., 2015). We adapted the JBI Manual for scoping review to fit the systematic search and review typology (Peters et al., 2015). Specifically, a health science librarian developed the search terms and strategies with feedback from the review team.

### 2.1 Study selection

#### 2.1.1 Information sources

The systematic search was conducted using four electronic databases: PubMed, Web of Science, CINAHL, and Embase. In addition to experimental and quasi-experimental studies, analytical observational studies, such as cross-sectional and prospective, and retrospective cohort studies, were considered for inclusion in this systematic scoping review. In addition, systematic reviews and clinical trial protocols were considered for inclusion to facilitate forward and backward citation searching.

#### 2.1.2 Search strategies

We presented the study eligibility criteria and search terms for the research question according to the Population, Intervention, Comparator, and Outcomes (PICO) framework (Schardt et al., 2007) in Table 1. Search limits were English language full text, human subjects, and the date of publication from January 1, 2004, to the time of the search. Databases are all searched on March 1st, 2024. The search terms were older adults (variations such as older adult, elderly, and specific age ranges were employed to capture the target population comprehensively), sleep (search terms covered various aspects of sleep, including patterns, quality, duration, and sleep disorders), balance (terms related to postural control, stability, and balance were included), and cognitive function (including memory, executive function, processing speed, and attention). The detailed search strategy is included in Supplementary Table 1.

**TABLE 1:** Population, intervention, comparator, and outcomes (PICO) description.

PICO elements	Description
Population	• Older adults
Intervention	• Any intervention that directly uses sleep, balance, or cognitive function as an intervention component • e.g., balance training, cognitive training, sleep therapies
Comparison	• Any group of older adults, whether they received alternative interventions, standard care, or no intervention, or have disease or conditions related to sleep, balance, and cognitive function • Or studies without a specific comparison group • e.g., quasi-experimental study with one group and observational study
Outcome	• Components related to sleep, balance, and cognitive function • e.g., sleep: sleep quality, duration, severity or prevalence of sleep disorders; balance: static and dynamic balance, balance confidence; cognitive function: cognitive function score, severity or prevalence of cognitive impairment

#### 2.1.3 Selection process

Covidence systematic review software (Veritas Health Innovation, Melbourne, Australia) was used to screen and select literature. Duplicates were automatically removed when they were imported into the Covidence web software. The literature screening process consisted of two phases: the title and abstract screening and the full-text screening. Studies were eligible for the full-text review if: (1) the average age of the study sample was 60 or over and (2) the outcome measures included two or more of the following: Sleep, cognitive function, motor balance, mobility, and related components of each, and (3) the study was human target research. Studies were excluded from further review if: (1) the type of study was conference abstracts, protocols, book reviews, or editorials, (2) the full text was not available, or (3) it was not written in English. The second inclusion criterion was revised for the full-text review to include studies that analyzed all three components. Studies were included only if they assessed all three domains (sleep, motor balance, and cognition) to ensure relevance to the review’s objective of synthesizing evidence on their triadic relationship. This strategy considered that some studies might not report all three components in the title and abstract but might report them in the full text. Detailed eligibility criteria for title/abstract screening and full-text review are in Supplementary Table 2.

We performed a calibration training of the title and abstract screening with 10 random references. In this test phase, inter-reviewer discussions were conducted to ensure mutual understanding of the eligibility criteria, which were then refined to enhance clarity for all reviewers. During the title/abstract screening phase, the relevance of each study based on the information provided in the title and abstract was assessed by a single first reviewer and one of the three independent second reviewers. Studies meeting the predefined inclusion criteria or those requiring further assessment based on ambiguity proceeded to the full-text screening phase. In the full-text screening phase, each eligible study underwent a thorough examination to determine its suitability for inclusion in the review. Any discrepancies between the reviewers’ assessments were resolved through consensus discussions. This rigorous screening process ensured the selection of studies that met the established criteria and contributed relevant data to the review.

### 2.2 Data extraction and analysis

Data extraction was carried out using a standardized extraction form by three reviewers. Extraction of information included: Article title, publication year, study site (country), research purpose, design, setting or data source, participant recruitment, participant eligibility criteria, study participant characteristics, sleep, motor balance, and cognitive function measurements, statistical analyses on the outcomes, and key findings. The extracted findings on sleep, motor balance, and cognitive function were narratively synthesized to identify the characteristics of each independent component and to explore the interaction characteristics of their combination within the older adult population.

### 2.3 Level of evidence of included studies

Each included study was assessed for its level of evidence using the guidelines from the National Health and Medical Research Council (NHMRC) (Merlin et al., 2009). The NHMRC scale categorizes study designs into four levels: Level I includes systematic reviews of randomized controlled trials (RCTs), offering the highest level of evidence due to a comprehensive analysis of multiple studies. Level II comprises individual RCTs, known for their rigorous design and control of bias. Level III is subdivided into three parts: III-1 includes pseudo-randomized controlled trials, III-2 encompasses cohort studies, case-control studies, and comparative studies with concurrent controls, and III-3 includes comparative studies with historical controls, single-arm studies, and interrupted time series without parallel controls. Level IV consists of case series, cross-sectional studies, and descriptive studies without control groups, providing the lowest level of evidence.

### 2.4 Risk of bias assessment

Risk of bias was assessed for all included studies using the Newcastle-Ottawa Scale (NOS) for observational studies and the Cochrane Risk of Bias 2 (RoB 2) tool for experimental studies. The NOS evaluates study quality based on three domains: selection, comparability, and outcome for case-control and cohort studies (Stang, 2010; Wells et al., 2025). For cross-sectional studies, a modified version of the NOS was applied to enable a more accurate assessment of bias (e.g., replaced follow-up-related items with assessment of statistical test appropriateness, reflecting the non-longitudinal nature of cross-sectional studies) (Herzog et al., 2013). The RoB 2 tool assesses risk of bias across five domains: the randomization process, deviations from intended interventions, missing outcome data, outcome measurement, and selection of the reported result (Sterne et al., 2019). One-group quasi-experimental studies were also evaluated using RoB 2, however, the randomization domain was excluded from the assessment. All evaluations were performed by Reviewer 1 and verified by Reviewer 2, with any disagreements resolved through discussion.

## 3 Results

### 3.1 Study selection

**FIGURE 1:**
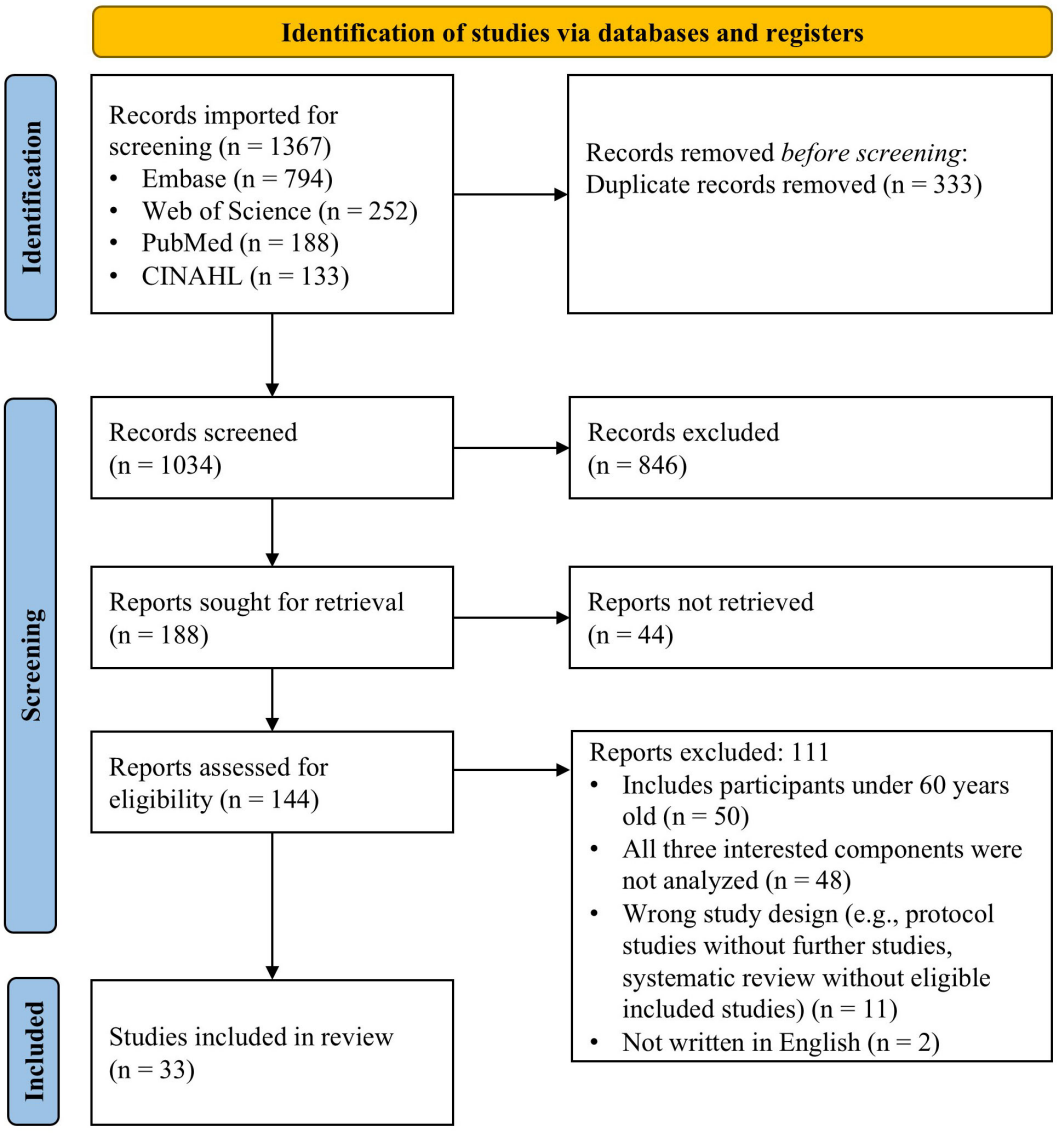
PRISMA flowchart summary of the literature search.

Initial searches of the databases (March 2024) returned 1,367 references, 333 of which were removed during duplicate screening. Title and abstract screening of the remaining 1,034 papers resulted in 144 papers remaining in the full-text screening phase. One hundred and eleven of these studies were excluded at the full-text phase (Figure 1), and a final 33 articles met inclusion criteria for the narrative synthesis. No additional studies were identified through forward and backward citation searching. This review aimed to collect studies that included sleep, motor balance, and cognitive function in order to identify patterns in the relationships between these three factors. Therefore, we included studies that included all three components in the target population, intervention, or outcome. For example, a study that compared motor balance and sleep between older adults with and without probable dementia was included in our review (Merlin et al., 2009). Although cognitive function was not directly measured as an outcome, the study analyzed differences in motor balance and sleep based on cognitive status, which met our inclusion criteria. Another example is a cross-sectional study that did not have an intervention or comparison group but assessed sleep quality, global cognition, and dynamic balance (Wells et al., 2025). This study was included because all three factors were assessed, and the relationships between them were analyzed.

### 3.2 Study characteristics

#### 3.2.1 Study designs

Among 33 studies, seven were experimental study designs: Four RCT studies and three quasi-experimental design studies (one group pretest-posttest). Twenty-six studies were observational study designs: Twenty cross-sectional designs, one prospective observational study, three prospective cohort studies, and two case-control studies. The descriptions of included studies, including the level of evidence based on the study design, are in Supplementary Tables 3, 4.

#### 3.2.2 Participant characteristics

The total analyzed sample size of the included studies was 67,237, and all participants in each study were 60 years old or over. Sixteen studies targeted older adults with specific diseases or health conditions: Mild cognitive impairment (MCI) or dementia (*n* = 5), diabetes (*n* = 2), Parkinson’s disease (PD; *n* = 3), and other conditions (*n* = 6; progressive supranuclear palsy, highly elevated blood cobalt, isolated rapid eye movement (REM) sleep behavior disorder, nursing home residents with core Lewy body dementia (LBD) sign, community-dwelling women with urinary incontinence, and chronic vestibular dysfunction). The other 17 studies were conducted targeting older adults in various settings without targeted health conditions: Community-dwelling older adults (*n* = 9), older adults residing in nursing homes (*n* = 2), healthy or physically active older adults (*n* = 2), and older adults who registered or attended health centers, clinics, or intervention programs (*n* = 4).

### 3.3 Outcome measurements

#### 3.3.1 Sleep measurements

The measured sleep components were classified into three domains. First, sleep quality was measured in 14 studies. Second, the prevalence or incidence of sleep-related conditions or symptoms was measured in 14 studies. Lastly, the severity of sleep-related conditions or symptoms was measured in seven studies. Thirty-one studies measured one of the three sleep domains, one study measured both the prevalence/incidence and severity of sleep-related conditions (Louis et al., 2016) and another study measured both sleep quality and the severity of sleep-related conditions (Scharre et al., 2016). The instruments and methods used to measure the sleep domains are listed in Supplementary Table 5.

#### 3.3.2 Motor balance measurements

Balance measurements were classified into balance performance, prevalence or incidence of sleep-related conditions, and balance confidence. If balance was measured without movement of the base of support, including assessment of postural sway, it was classified as static balance and measured in eight studies. When balance was measured with movement of the base of support, it was classified as dynamic balance and measured in four studies. Measurements that assessed static and dynamic balance were classified as overall balance and measured in thirteen studies. The prevalence or incidence of sleep-related conditions was measured in seven studies, and balance confidence was measured in one study. The instruments and methods used to measure the balance domains are listed in Supplementary Table 6.

#### 3.3.3 Cognition measurements

The cognition measurements used in each study were classified into global cognition, cognitive function subcomponents, and patient-reported clinical symptoms of cognition. Global cognition was measured in 27 studies, and individual measurements of specific cognitive components were also conducted: Memory (*n* = 5), executive function (*n* = 4), visuospatial function (*n* = 3), verbal fluency (*n* = 2), structural language skills (*n* = 1), orientation (*n* = 1), and confrontation naming ability (*n* = 1). Finally, patient-reported clinical symptoms of cognition were measured in nine studies. The instruments and methods used to measure the cognition domains are listed in Supplementary Table 7.

### 3.4 Relations between Sleep, motor Balance, and cognition

All 33 studies conducted statistical analyses on sleep, balance, and cognitive function and reported the three outcomes. The findings were classified into three different types. First, type A studies reported independent findings on sleep, balance, and cognitive function without examinations among the three outcome measures (*n* = 24) (Supplementary Tables 3, 4). Second, type B studies reported either a direct or indirect relationship between two of the three outcomes of sleep, balance, and cognitive function (*n* = 9). Target population, assessments, statistical values, significance/effect size of the interactions, and interpreted interaction between domains are described in Tables 2, 3. These tables summarize direct correlations and associations and group comparisons in sleep, motor, and cognition, categorized by pairwise relationships and group differences, respectively. Statistical values include Spearman’s correlation coefficients (r_s_), odds ratios (OR), beta coefficients (β) with standard errors (SE), F-statistics, means with 95% confidence intervals (CI), medians, and percentages, interpreted per Cohen’s guidelines (e.g., *d* < 0.2: very weak; 0.2–0.5: weak; 0.5–0.8: medium; >0.8: large) for clinical relevance (Cohen, 1988; Sullivan and Feinn, 2012). Third, the type C studies reported relationships among all three measures. Only one study indirectly analyzed the associations among all three measures.

**TABLE 2:** Correlations and associations among sleep, motor balance, and cognition.

References	Study design (LoE)	Target population	Statistical analysis	Domains and measurements	Statistical value	Interaction interpretation
**Sleep and motor balance**						
Schnittger et al., 2012	Cross-sectional (IV)	Community-dwelling older adults	Spearman’s rank correlation	Sleep quality (PSQI factor score) – dynamic balance (TUG)	r_s_ = −0.139*	Very weak positive correlation
				Sleep quality (PSQI factor score) – overall balance (BBS)	r_s_ = 0.178**	Very weak positive correlation
Ganidagli et al., 2023	Cross-sectional (IV)	Older adults in geriatric clinic	Spearman’s rank correlation	Sleep quality (PSQI) – overall balance (POMA)	r_s_ = −0.263*	Weak positive correlation
Paker et al., 2018	Cross-sectional (IV)	Community-dwelling older adults in clinics	Multivariable logistic regression	Insomnia prevalence (self-report) – balance confidence (ABC scale)	OR = 0.325***	Strong negative association
Catikkas et al., 2023	Cross-sectional (IV)	Older adults with diabetes	Multivariable logistic regression	Prevalence of EDS (ESS < cut-off) – overall balance (POMA)	OR = 0.956*	Very weak negative association
**Sleep and cognition**						
Schnittger et al., 2012	Cross-sectional (IV)	Community-dwelling older adults	Spearman’s rank correlation	Sleep quality (PSQI factor score) – global cognition (MMSE)	r_s_ = 0.157*	Very weak positive correlation
Ganidagli et al., 2023	Cross-sectional (IV)	Older adults in geriatric clinic	Spearman’s rank correlation	Sleep quality (PSQI) – global cognition (MMSE)	r_s_ = −0.082	Very weak, insignificant positive correlation
Chen et al., 2020	Prospective cohort (II)	Older adults with MCI	Binary logistic regression	Sleep quality (PSQI > 5) – incidence of cognitive decline (MMSE > 2 points decline)	β (SE) = −4.710 (3.056)	Strong, insignificant negative association
Catikkas et al., 2023	Cross-sectional (IV)	Older adults with diabetes	Multivariable logistic regression	Prevalence of EDS (ESS < cut-off) – global cognition (MMSE)	OR [95% CI] = 0.926 [0.875–0.981]	Very weak negative association
				Prevalence of EDS (ESS < cut-off) – dementia diagnosis (CGA-dementia)	OR [95% CI] = 2.457 [1.260–4.792]	Moderate positive association
**Motor balance and cognition**						
Ganidagli et al., 2023	Cross-sectional (IV)	Older adults in geriatric clinic	Spearman’s rank correlation	Overall balance (POMA) – global cognition (MMSE)	r_s_ = 0.054	Very weak, insignificant positive correlation
Paker et al., 2018	Cross-sectional (IV)	Community-dwelling older adults in clinics	Multivariable logistic regression	Balance confidence (ABC score > 67) – global cognition (MMSE)	OR = 1.124***	Very weak positive association
Scharre et al., 2016	Cross-sectional (IV)	Older adults with LBD, AD, or PD	Spearman’s rank correlation	Overall balance (BBS) – global cognition (SAGE)	r_s_ = 0.574***	Moderate positive correlation
				Overall balance (BBS) – global cognition (MMSE)	r_s_ = 0.436	Weak, insignificant positive correlation
				Overall balance (BBS) – global cognition (CERAD)	r_s_ = 0.503***	Moderate positive correlation
				Overall balance (POMA) – global cognition (SAGE)	r_s_ = 0.492	Weak, insignificant positive correlation
				Overall balance (POMA) – global cognition (MMSE)	r_s_ = 0.393	Weak, insignificant positive correlation
				Overall balance (POMA) – global cognition (CERAD)	r_s_ = 0.443	Weak, insignificant positive correlation
				Overall balance (UPDRS III) – global cognition (SAGE)	r_s_ = −0.401	Weak, insignificant positive correlation
				Overall balance (UPDRS III) – global cognition (MMSE)	r_s_ = −0.296	Weak, insignificant positive correlation
				Overall balance (UPDRS III) – global cognition (CERAD)	r_s_ = −0.344	Weak, insignificant positive correlation
				Overall balance (BBS) – executive function (SAGE executive)	r_s_ = 0.582***	Moderate positive correlation
				Overall balance (POMA) – executive function (SAGE executive)	r_s_ = 0.520***	Moderate positive correlation
				Overall balance (UPDRS III) – executive function (SAGE executive)	r_s_ = −0.377	Weak, insignificant positive correlation
				Overall balance (BBS) – visuospatial (SAGE visuospatial)	r_s_ = 0.420	Weak, insignificant positive correlation
				Overall balance (POMA) – visuospatial (SAGE visuospatial)	r_s_ = 0.574	Moderate, insignificant positive correlation
				Overall balance (UPDRS III) – visuospatial (SAGE visuospatial)	r_s_ = −0.220	Weak, insignificant positive correlation
				Overall balance (BBS) – memory (CERAD memory)	r_s_ = 0.342	Weak, insignificant positive correlation
				Overall balance (POMA) – memory (CERAD memory)	r_s_ = 0.278	Weak, insignificant positive correlation
				Overall balance (UPDRS III) – memory (CERAD memory)	r_s_ = −0.240	Weak, insignificant positive correlation
				Overall balance (BBS) – verbal fluency (FAS)	r_s_ = 0.427	Weak, insignificant positive correlation
				Overall balance (POMA) – verbal fluency (FAS)	r_s_ = 0.430	Weak, insignificant positive correlation
				Overall balance (UPDRS III) – verbal fluency (FAS)	r_s_ = −0.352	Weak, insignificant positive correlation

ABC, activities-specific balance confidence; AD, Alzheimer’s disease; β, beta coefficients; BBS, Berge Balance Scale; CERAD, consortium to establish a registry for Alzheimer’s disease; CGA, comprehensive geriatric assessment; CI, confidence interval; EDS, excessive daytime sleepiness; ESS, Epworth sleepiness scale; LBD, Lewy body dementia; LoE, level of evidence; MCI, Mild cognitive impairment; MMSE, Mini-Mental State Examination; OR, odd ratio; PD, Parkinson’s disease; POMA, Tinetti Performance Oriented Mobility Assessment; PSQI, Pittsburgh Sleep Quality Index; SAGE, self-administered gerocognitive examination; r_s_, Spearman’s correlation coefficient; SE, standard error; TUG, Timed-Up and Go; UPDRS, unified Parkinson’s disease rating scale.

*, *p* < 0.05;

**, *p* < 0.01;

***, *p* < 0.001.

**TABLE 3:** Group differences in sleep, motor balance, and cognition.

References	Study design (LoE)	Groups	Statistical analysis	Domains and measurements	Statistical value	Interaction interpretation
**Grouped based on sleep**						
Vogel et al., 2021	RCT (II)	Exercise group with recommended sleep duration vs. short/excessive	Analysis of covariance	Dynamic balance (functional reach test)	*F* = 0.220 (η^2^_p_ = 0.016)	No difference (small effect)
				Global cognition (MoCA)	*F* = 5.498*** (η^2^_p_ = 0.287)	More improved in recommended duration (large effect)
Nisser et al., 2022	Cross-sectional (IV)	With iRBD vs. without	Multivariate ANCOVA	Dynamic balance (SLWT-In line walk forward 20 cm)	Means [95% CI] = 0.2 [−0.0;0.4] vs. 0.0 [−0.2;0.2]	No difference
			Multivariate ANCOVA	Dynamic balance (SLWT-in line walk forward 15 cm)	Means [95% CI] = 0.7 [0.2;1.2] vs. 0.0 [−0.4;0.4]	No difference
			Multivariate ANCOVA	Dynamic balance (SLWT-in line walk backward 20 cm)	Means [95% CI] = 1.8 [1.2;2.4] vs. 0.6 [0.0;1.2]*	Lower in iRBD
			Multivariate ANCOVA	Dynamic balance (SLWT-in line walk backward 15 cm)	Means [95% CI] = 3.3 [2.2;4.3] vs. 1.1 [0.1;2.1]*	Lower in iRBD
			ANCOVA	Dynamic balance (TUG)	Means [95% CI] = 7.5 [6.7;8.3] vs. 7.2 [6.5;7.9]	No difference
			Multivariate ANCOVA	Center of pressure at the parallel stand (force plate)	Means [95% CI] = 196.1 [165.1;227.1] vs. 208.5 [182.5;234.6]	No difference
			Multivariate ANCOVA	Center of pressure at the standing on one leg, right (force plate)	Means [95% CI] = 1252.8 [920.5;1585.1] vs. 900.1 [621.0;1179.0]	No difference
			Multivariate ANCOVA	Center of pressure at the standing on one leg, left (force plate)	Means [95% CI] = 1214.1 [849.7;1578.6] vs. 985.6 [679.1;1292.1]	No difference
			*t*-test	Global cognition (MMSE)	Mean [95% CI] = 27.5 [25.9;29.0] vs. 28.6 [27.8;29.4]	No difference
Catikkas et al., 2023	Cross-sectional (IV)	With EDS vs. without	Mann–Whitney U test	Overall balance (POMA)	Median = 20 (0–28) vs. 26 (0–28)** (Rosenthal’s *r* = 0.27)	Lower in with EDS (small effect)
			*t*-test	Global cognition (MMSE)	Mean (SD) = 20.4 (6.9) vs. 22.9 (4.9)* (*d* = 0.41)	Lower in with EDS (medium effect)
			Chi-square	Dementia diagnosis (CGA-dementia)	*n* (%) = 22 (48.9%) vs. 51 (28%)** (OR = 2.45)	More in with EDS (medium effect)
**Grouped based on motor balance**						
Paker et al., 2018	Cross-sectional (IV)	Lower balance confidence vs. higher	Chi-square	Insomnia prevalence (self-report)	*n* (%) = 42 (71.2%) vs. 45 (44.6%) (OR = 2.85)	Higher rate in lower confidence (medium effect)
			Mann–Whitney U test	Global cognition (MMSE)	Mean (SD) = 19.1 (4.9) vs. 21.9 (4.8) (*d* = 0.58)	Lower in lower confidence (medium effect)
**Grouped based on cognition**						
Amjad et al., 2019	Cross-sectional (IV)	With probable dementia vs. without	Chi-square	Prevalence of insomnia (self or proxy report)	Percentage difference shown in graph	No difference
				Prevalence of poor balance/coordination (self or proxy report)	Percentage difference shown in graph***	More in probable dementia
Chen et al., 2020	Prospective cohort (II)	PD-MCI with cognitive decline vs. without	Chi-square, *t*-test	Sleep quality (PSQI > 5)	*n* (%) = 8 (88.9%) vs. 8 (36.4%)** (OR = 8.5)	Lower in cognitive decline (large effect)
				Dynamic balance (TUG)	Mean = 28.69 vs. 16.34	No difference
				Dynamic balance (TUG-sit to stand)	Mean = 2.17 vs. 1.84	No difference
				Dynamic balance (TUG-mid turning)	Mean = 4.06 vs. 2.65	No difference
				Dynamic balance (TUG-end turning)	Mean = 4.20 vs. 2.48	No difference
				Dynamic balance (TUG-stand to sit)	Mean = 2.97 vs. 2.37	No difference
				Dynamic balance (POMA-balance)	Mean = 13.00 vs. 14.62	No difference

ANCOVA, analysis of covariance; CGA, comprehensive geriatric assessment; CI, confidence interval; EDS, excessive daytime sleepiness; iRBD, isolated rapid eye movement sleep behavior disorder; LoE, level of evidence; MCI, mild cognitive impairment; MMSE, Mini-Mental State Examination; MoCA, Montreal Cognitive Assessment; OR, odds ratio; PD, Parkinson’s disease; POMA, Tinetti Performance Oriented Mobility Assessment; PSQI, Pittsburgh Sleep Quality Index; SD, standard deviation; SLWT, straight line walk test; TUG, Timed-Up and Go; η^2^_p_, Partial eta squared.

*, *p* < 0.05;

**, *p* < 0.01;

***, *p* < 0.001.

#### 3.4.1 Relationship between sleep and motor balance

Four studies directly examined correlations or associations among the six studies that reported the relationship between sleep and motor balance. Sleep quality and overall balance showed significant weak positive associations (*r* = 0.1–0.3, per Cohen’s guidelines), with one study indicating a significant weak negative correlation between Pittsburgh Sleep Quality Index (PSQI) score and Tinetti Performance Oriented Mobility Assessment (POMA) score (*r* = −0.263; *p* < 0.05) (Ganidagli et al., 2023) and another showing a significant positive correlation between the PSQI factor score and Berge Balance Scale (BBS) score (*r* = 0.178; *p* < 0.01) (Schnittger et al., 2012). The relationship between sleep quality and dynamic balance was examined in one study, which found a significant weak negative correlation between the PSQI factor score and Timed-Up and Go (TUG) test (*r* = −0.139; *p* < 0.05) (Schnittger et al., 2012), suggesting a positive relationship between sleep quality and dynamic balance. Lastly, the prevalence of sleep problems increased with lower balance confidence, measured by the Activities-specific Balance Confidence (ABC) scale, and overall balance performance, measured by the POMA (Catikkas et al., 2023; Paker et al., 2018). The relationship between sleep and motor balance was also indirectly reported in four studies by comparing differences in motor balance and sleep between groups based on the presence or absence of sleep or motor balance conditions. Dynamic balance performance among older adults was lower in those with isolated REM sleep behavior disorder (iRBD) than in those without (Nisser et al., 2022). At the same time, no significant differences were found between recommended sleep duration and insufficient or excessive sleep duration (Vogel et al., 2021). Another study reported that overall balance was poorer in those with excessive daytime sleepiness (EDS) (Catikkas et al., 2023), and one other study found a higher prevalence of insomnia in those with lower balance confidence (Paker et al., 2018).

#### 3.4.2 Relationship between sleep and cognition

The relationship between sleep and cognition was reported in 7 studies. Four studies directly analyzed the correlation and association between sleep and cognition. The relationship between sleep quality and global cognition showed a significant weak positive correlation between the PSQI factor score and Mini-Mental State Examination (MMSE) (*r* = 0.157, *p* < 0.05) in one study (Herzog et al., 2013). In contrast, another found a non-significant minimal correlation between PSQI and MMSE (*r* = −0.082) (Sterne et al., 2019). In one study, sleep quality, measured by the PSQI, was not significantly associated with the prevalence of cognitive decline (Cohen, 1988). A higher prevalence of sleep problems was associated with a higher prevalence of dementia and lower global cognition, measured by MMSE, in another study (Catikkas et al., 2023). In group difference analysis, older adults with EDS had more dementia diagnoses compared to those without EDS (Catikkas et al., 2023). However, global cognition between older adults with iRBD and those without iRBD showed no difference (Nisser et al., 2022). Furthermore, a higher proportion of older adults with cognitive problems had poor sleep quality compared to those without cognitive problems (Cohen, 1988), although there was no difference in the prevalence of insomnia (Amjad et al., 2019). Lastly, the effect of exercise intervention on global cognition was higher in older adults with recommended sleep duration compared to those with insufficient or excessive sleep duration (Vogel et al., 2021).

#### 3.4.3 Relationship between motor balance and cognition

Two studies reported a correlation between motor balance and cognition, and 22 reports of the correlations were collected. Among the 10 correlation reports on overall balance and global cognition, 2 showed significantly strong associations (*r* > 0.5), while the other 8 reports showed non-significant weak to strong associations (*r* = 0.054–0.574). Among the 3 correlation reports on overall balance and executive function, 2 showed significant strong associations (*r* > 0.5). Global cognition, measured by MMSE, showed a positive association with increased balance confidence (ABS score > 67) (Paker et al., 2018). In addition, individuals with cognitive problems had more motor balance problems compared to those without cognitive problems (Amjad et al., 2019), but there was no significant difference in dynamic balance performance, measured by the TUG and motor balance test in POMA (Chen et al., 2020).

#### 3.4.4 Relationship between sleep, motor balance, and cognition

Only one study analyzed the indirect interaction among all three components. The significant difference in overall balance between older adults with EDS and those without EDS remained significant after controlling for the presence of dementia (Ganidagli et al., 2023).

#### 3.4.5 Medication data and analytical limitations

Of the nine studies that analyzed dyadic or triadic associations between sleep, motor balance, and cognition, only five reported information about participants’ medication use in their eligibility criteria or demographic data (Catikkas et al., 2023; Ganidagli et al., 2023; Nisser et al., 2022; Paker et al., 2018; Scharre et al., 2016). Individuals with sleep-related symptoms (e.g., poor sleep quality, EDS, or iRBD) were found to use more medications than those without such symptoms (Catikkas et al., 2023; Ganidagli et al., 2023; Nisser et al., 2022). Additionally, participants with lower balance confidence used more medications than those with higher balance confidence (Paker et al., 2018). The LBD group used fewer anti-Parkinsonian medications than the PD group, but more than the AD group (Paker et al., 2018; Scharre et al., 2016). Only the study by Catikkas et al. (2023) explicitly excluded participants taking medications for sleep problems. Ganidagli et al. (2023) analyzed the association between the number of medications and sleep quality; however, the result was not statistically significant (PSQI; *r* = 0.10, *p* = 0.12). The remaining studies acknowledged the potential influence of medication use but did not include it in their analyses on sleep, motor balance, and cognition.

#### 3.4.6 Statistical methods and synthesis challenges

Among the 33 studies, the relationships among sleep, motor balance, and cognition were identified in 9 studies through varied statistical analyses. The methods used included correlations (*n* = 3), regressions (*n* = 3), and group comparisons (*n* = 6). Correlational studies primarily reported weak to moderate associations (*r* = 0.05–0.58), while odds ratios indicated an increased risk of balance and cognitive impairments among individuals with sleep problems. These differences complicated direct comparisons (see Tables 2, 3 for details). Moderators such as age, sex, and disease status were reported inconsistently across studies. Disease status (e.g., PD) was a significant moderator in 10 studies, while age and sex showed variable effects in 5 studies each, limiting comprehensive analysis.

#### 3.4.7 Risk of bias of included studies

Risk of bias was assessed for the 33 included studies (Supplementary Figure 1 and Supplementary Table 9). The original NOS tool does not provide standardized criteria for categorizing the overall risk of bias. However, in this review, all five cohort or case-control studies scored 7 or higher out of a maximum of 9 points. Similarly, among the 21 cross-sectional studies, all scored 7 or higher out of 10 points, with one study scoring 6. Among the four RCTs, all were rated as having “some concerns” according to the RoB 2 tool. The three included one-group quasi-experimental studies were rated as low risk based on RoB 2. A common issue identified in the RCTs was insufficient reporting on allocation concealment and blinding.

## 4 Discussion

This systematic scoping review synthesized evidence from 33 studies to elucidate the interrelationships among sleep, motor balance, and cognition in older adults. Acceptable risk of bias was observed in the majority of studies, with 26 observational studies rated above half of the total scores in NOS and NOS for cross-sectional studies. However, a common limitation among cross-sectional studies was the “lack of information regarding non-respondents.” Similarly, the seven experimental studies, including four RCTs and three one-group quasi-experimental designs, also demonstrated an acceptable level of bias. Notably, all four RCTs failed to report allocation concealment, raising concerns about potential selection bias. A consistent pattern of positive associations emerged between pairs of these factors:

### 4.1 Sleep and motor balance

Reduced sleep problem prevalence and sleep quality correlated with improved motor balance performance and confidence, aligning with prior reviews (Chen et al., 2020; Paker et al., 2018; Schnittger et al., 2012; Umemura et al., 2022). The positive correlation between overall and dynamic balance with sleep quality also aligns with prior findings that. This bidirectional relationship suggests that interventions targeting either domain could yield dual benefits, particularly in populations like older adults with LBD who experience concurrent sleep and balance impairments (Soysal et al., 2023; Zahirovic et al., 2019).

### 4.2 Sleep and cognition

A negative correlation between sleep problem prevalence and global cognition (Ganidagli et al., 2023), contrasted with an indirect positive link to cognitive problem prevalence, underscores sleep’s protective role in cognitive health. These findings reinforce the importance of sleep-related interventions to preserve cognitive function (Qin et al., 2023; Sakal et al., 2024). However, positive correlation between sleep quality and global cognition was only significant in one study suggesting that the presence of actual sleep problems and an individual’s perceived sleep quality may be differently associated with cognitive function (Sullivan and Feinn, 2012).

### 4.3 Motor balance and cognition

Balance confidence is positively correlated with global cognition (Paker et al., 2018), while static/dynamic balance is linked to cognitive subcomponents like memory and processing speed (Divandari et al., 2023). However, mixed results in subcomponent analyses (e.g., executive function vs. overall balance) highlight the need for tailored approaches in conditions like Alzheimer’s disease and PD (Chen et al., 2020; Scharre et al., 2016).

Although several studies found statistically significant correlations between sleep, balance, and cognitive measures, most of these were weak. Weak correlations (e.g., *r* = 0.139) may have limited clinical significance at the individual level but could be relevant in large populations, such as for public health screening. Moderate, non-significant correlations (e.g., *r* = 0.574) suggest potential relationships that warrant further investigation with larger samples. Consistent reporting of effect sizes in future studies is critical to clarify clinical impact.

**FIGURE 2:**
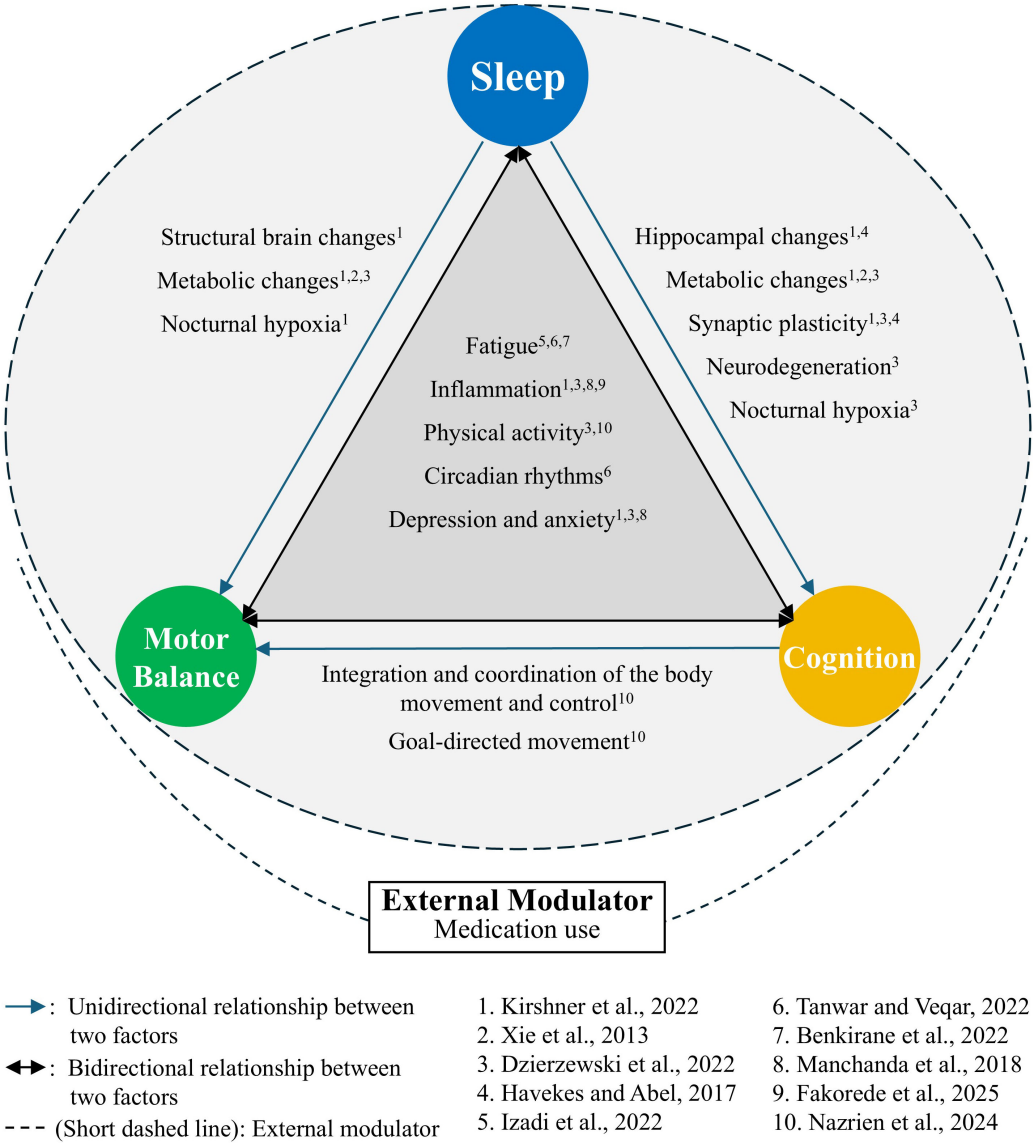
Hypothesized model of triadic relationships among sleep, motor balance, and cognition in older adults: Sleep (including sleep quality and problems) influences motor balance (overall, dynamic, and confidence aspects) and cognition (global, executive, and impairment aspects) via shared mechanisms. Fatigue, inflamation, physical activity, circadian rhythms, depression, and anxiety are factors that influence sleep, motor balance, and cognition, and that are, in turn, influenced by them. Consequently, these factors may directly or indirectly account for the observed relationships among those three domains (Benkirane et al., 2022; Dzierzewski et al., 2022; Kirshner et al., 2022; Nazrien et al., 2024; Tanwar and Veqar, 2022). Bidirectional arrows indicate reciprocity of the three domains. Sleep has direct and indirect effects on motor balance and cognition. For example, inadequate clearance of metabolic waste due to insufficient sleep caused by sleep disorders can lead to neural dysfunction, impairing motor balance and cognitive function (Kirshner et al., 2022; Xie et al., 2013). The relationships between motor balance and cognition is generally conceptualized as changes in cognition leading to changes in balance (Yu et al., 2021). Cognitive functions, including memory, attention, executive function, and visual-spatial processing, can influence the integration and coordination of body movements and the performance of goal-directed movements, thereby affecting motor balance (Nazrien et al., 2024). While previous clinical trials have indicated that cognitive training can improve motor balance and that balance training can enhance cognitive function, research exploring the underlying mechanisms of these changes is limited. Further investigation into the potential mechanism (e.g., decline in motor balance reduces physical accessibility, thereby limiting opportunities for learning and cognitive stimulation) is needed. External modulators, like medication use, may influence these pathways. This framework guides future multimodal interventions targeting the triad.

Notably, only one study explored the triadic relationship among sleep, motor balance, and cognition, highlighting a critical gap in the literature, reporting an insignificant mediation effect of cognition on sleep-balance associations (Catikkas et al., 2023). To elucidate the relationships among sleep, motor balance, and cognition, we propose a mechanistic framework integrating clinical and pre-clinical evidence. Sleep, motor balance, and cognition likely interact through shared neural pathways, notably the prefrontal cortex, basal ganglia, and hippocampus, which regulate executive function, motor control, and memory consolidation. Pre-clinical studies in rodents show that sleep deprivation impairs hippocampal cAMP-PKA signaling, reducing synaptic plasticity and spatial memory performance (Havekes and Abel, 2017). Similarly, chronic sleep restriction in mice increases neuroinflammation via pro-inflammatory cytokines (e.g., TNF-α, IL-6), contributing to cognitive deficits (Manchanda et al., 2018). Sleep also supports glymphatic clearance of metabolic waste, and its disruption may exacerbate neural dysfunction affecting motor and cognitive performance (Xie et al., 2013). In older adults, these effects are amplified by age-related declines in neural plasticity. Fatigue and neuroinflammation may mediate these interactions, as sleep loss impairs postural control and attention (Izadi et al., 2022). Although studies directly explaining the triadic relationship are limited, overlapping mechanisms among proposed mechanisms for dyadic relationships can help infer plausible mechanisms underlying the triadic relationship (Figure 2; Benkirane et al., 2022; Dzierzewski et al., 2022; Fakorede et al., 2025; Izadi et al., 2022; Kirshner et al., 2022; Manchanda et al., 2018; Nazrien et al., 2024; Tanwar and Veqar, 2022; Xie et al., 2013; Yu et al., 2021).

Population heterogeneity among the included studies significantly influenced the observed associations among sleep, motor balance, and cognition. In older adults with neurodegenerative conditions (e.g., PD, LBD), prominent motor balance impairments aligned closely with motor dysfunction, whereas relationships involving sleep varied depending on cognitive trajectory (Scharre et al., 2016). Specifically, in PD and MCI, deteriorating sleep quality was particularly evident among individuals experiencing cognitive decline. Similarly, those with LBD or PD exhibited selective associations, notably significant correlations between executive function and overall balance performance. Older adults with sleep-related conditions, such as iRBD or EDS, demonstrated specific impairments in dynamic balance, particularly during complex motor tasks, alongside global cognitive deficits (Nisser et al., 2022). Conversely, in non-neurological populations, including community-dwelling older adults and those with diabetes, associations were less consistent, predominantly emerging as correlations between sleep quality or EDS and overall balance or cognitive function. Additionally, healthy older adults with fewer comorbidities showed weaker or minimal associations, potentially due to a lower burden of underlying pathologies (Supplementary Tables 3, 4).

Medications are potential confounders in the relationships among sleep, motor balance, and cognition in older adults, yet their impact is underexplored in our review. Five of the nine included studies that analyzed the associations between sleep, motor balance, and cognition reported medication use. However, none of these studies analyzed the impact of medication use on the associations between each component, and only two studies specified which medications were used, limiting the synthesis of their effects. Sedatives and antipsychotics, which target neurotransmitter systems (e.g., GABA, dopamine), may disrupt sleep architecture and impair cognitive function or motor balance. For instance, sedative use is associated with increased fall risk and cognitive decline in older adults (Stone et al., 2014; Suh et al., 2018). Antihypertensives, such as statins, may influence cognition via vascular mechanisms, with mixed effects on cognitive performance (Trompet et al., 2010). These findings suggest medications could mediate or confound the sleep–motor balance–cognition triad, potentially exacerbating age-related declines in neural plasticity. Future longitudinal studies should control for medication use, employing standardized reporting (e.g., medication type, dosage) to clarify their role in these interactions. Physical activity (e.g., Vogel et al., 2021) and depression (e.g., Schnittger et al., 2012) were noted as potential mediators or confounders in some studies but were not consistently analyzed. Physical activity may enhance motor balance and cognition, while depression could exacerbate sleep disturbances. Future studies should control these variables to clarify their roles in the sleep-balance-cognition triad.

The review’s limitations highlight critical gaps in the current evidence base. First, there was limited triadic research. Only one out of 33 studies examined sleep, motor balance, and cognition together, while the other nine explored these relationships in pairs. These limitations of direct triadic analyses limit the conclusions that can be drawn about how these domains interact holistically. Future reviews could explore these associations to complement our findings. Secondly, there was heterogeneity in the tools used across different studies. Varied assessment tools (e.g., BBS vs. POMA for motor balance, differing cognitive subcomponent measures) and population diversity (e.g., skewed sex ratios, comorbid conditions) complicated cross-study comparisons (Morris et al., 2018; Scharre et al., 2016). To enhance comparability, future studies should adopt standardized tools with validated psychometric properties, such as the Montreal Cognitive Assessment (MoCA) for cognition, BBS for motor balance, and the PSQI for sleep. These tools offer robust reliability and sensitivity to age-related changes (Supplementary Table 8). Thirdly, over half of the included studies were cross-sectional (19/26), precluding causal inferences. Also, a meta-analysis was not feasible due to heterogeneity in study designs, populations, and outcome measures, consistent with the exploratory aim of scoping reviews per JBI guidelines.

The findings underscore the interconnectedness of sleep, motor balance, and cognition, advocating for integrated intervention strategies. For example, motor balance training programs might concurrently enhance sleep quality and cognitive function in older adults. However, the review identifies critical priorities for future research: (1) Longitudinal studies are needed to determine causal relationships and mediation effects among the three domains. (2) Using uniform assessment tools (e.g., standardized motor balance tasks and cognitive subcomponent definitions) will improve comparability across studies. (3) Investigating these interactions in cohorts with neurodegenerative diseases (e.g., LBD, Alzheimer’s) could inform more targeted therapies. Our ongoing 6-weeks observational study examines the relationships among sleep (measured daily via smart ring and sleep diaries), balance (assessed weekly using the smart balance board and functional balance assessment), and cognition (assessed weekly via NIH Toolbox) in community-dwelling older adults. Building on this, we are planning a 12-months longitudinal trial to elucidate causal pathways in the sleep-motor balance-cognition triad. The trial would combine three evidence-based interventions: Tai Chi to enhance motor balance by improving postural stability (Liu and Frank, 2010), cognitive training to bolster executive function and memory (Hill et al., 2017), and sleep hygiene education to improve sleep quality (Chung et al., 2018). Over 12 months, participants would engage in weekly Tai Chi sessions, biweekly cognitive training (e.g., computer-based tasks), and monthly sleep hygiene workshops. Standardized measures, including the PSQI and sleep parameters from wearables, BBS, and NIH Toolbox cognition, would assess outcomes at baseline, 6, and 12 months. Mediation analyses would explore causal pathways, testing whether improved sleep quality mediates balance and cognitive gains, or if enhanced balance confidence reduces anxiety, improving sleep and cognition. Controlling for confounders like medication use and physical activity (tracked by the wearables) is critical. This trial would provide robust evidence for integrated interventions, informing clinical strategies to maintain functional independence in aging populations.

## 5 Conclusion

While this review identifies positive pairwise relationships among sleep, motor balance, and cognition, the holistic dynamics of the triad remain underexplored. The integration of existing evidence lays a foundation for recognizing reciprocal interactions but calls for rigorous, multidisciplinary studies to disentangle mechanisms and optimize interventions. Future research should prioritize longitudinal designs to explore the dynamic interplay of sleep, motor balance, and cognition, informing targeted interventions for older adults. As further research accumulates, especially with longitudinal and methodologically robust designs, it will pave the way for effective, integrated intervention and management strategies aimed at mitigating functional decline and enhancing the quality of life for older adults. To address identified gaps, we propose a longitudinal study with multi-month or year follow-ups, using standardized measures (e.g., MoCA, BBS, PSQI) and mediation analyses to explore causal pathways. We aim to collaborate with research networks to pursue such studies, enhancing our understanding of the sleep-balance-cognition triad.

## Data Availability

The original contributions presented in this study are included in this article/Supplementary material, further inquiries can be directed to the corresponding author.
