# Supplementary material for: Mapping the relationships among sleep, motor balance, and cognition in older adults: a systematic scoping review
Source: Front Hum Neurosci. 2025 Sep 1;19:1575155. doi: 10.3389/fnhum.2025.1575155 (PMC12433963; doi:10.3389/fnhum.2025.1575155)
Supplement: Supplementary file 1 [file Data_Sheet_1.docx]

Table A1. Search Strategy

| Databases | PubMed, Web of Science, CINAHL, Embase |
| --- | --- |
| Keywords | - Older adults - Cognitive function - Balance (or mobility) - Sleep |
| Search limits | - Search dates: January 2004 – date of search - Restrictions: English language and human study - Evidence type: Filter out conference abstracts, protocols, book reviews, editorials |
| Search terms | 1. Older adults   Title and abstract  "older adult*" OR "old adult*" OR elder* OR "old people" OR "older people" OR "old person" OR "older person" OR "older communit*" OR "older population*" OR "senior people" OR "senior person" OR "senior citizen*" OR "senior communit*" OR "senior population*" OR geriatric* OR "oldest old" OR "old age" OR "old aged" OR "older age" OR "older aged" OR "over 65" OR "over 60" OR "over 70" OR "over 75" OR "over 80" OR "over 85" OR "over 90" OR "over 95" OR "over 6*" OR "over 7*" OR "over 8*" OR "over 9*"  + Subject heading (aged)   1. Sleep   Title and abstract  Sleep OR “sleep pattern” OR “sleep quality” OR “sleep duration” OR “sleep continuity” OR “sleep fragmentation” OR “sleep architecture” OR “sleep disturbance” OR “sleep disorder” OR insomnia OR “sleep deprivation” OR “sleep apnea” OR “restless legs syndrome” OR RLS OR narcolepsy OR parasomnia OR “circadian rhythm disorders” OR hypersomnia OR “sleep-wake disorders”  + Subject heading (sleep and sleep disorder)   1. Balance   Title and abstract  balance OR “static balance” OR “dynamic balance” OR “postural control” OR “posture equilibriums” OR “posture balance” OR “posture control”  + Subject heading (postural balance and core stability)   1. Cognitive function   Title and abstract  cognition OR “cognitive*” OR “cognitive ability” OR “cognitive function” OR memory OR “episodic memory” OR “declarative memory” OR “short-term memory” OR “long-term memory” OR “working memory” OR “verbal memory” OR “spatial memory” OR “object memory” OR “location memory” OR encoding OR storage OR retrieval OR “free recall” OR “cued recall” OR “forced-choice recognition” OR recall OR “procedural memory” OR “semantic memory” OR “prospective memory” OR “executive function” OR “executive functioning” OR reasoning OR “problem-solving” OR processing OR “processing speed” OR fluency OR coding OR tracking OR execution OR executive OR maintenance OR manipulation OR attention OR concentration OR selective attention OR “sustained attention” OR vigilance OR multisensory OR perception OR “object recognition” OR “organizational strategies” OR naming OR reading OR comprehension OR mental OR arousal  + Subject heading (cognition, cognitive disorder, mental processes) |
| Basic search strategy | ("older adults"[Title/Abstract]) AND ("cognitive function"[Title/Abstract]) AND (sleep[Title/Abstract]) AND (balance[Title/Abstract]) |
| The actual searching term in each database | - PubMed   ("older adult*"[Title/Abstract] OR "old adult*"[Title/Abstract] OR elder*[Title/Abstract] OR "old people"[Title/Abstract] OR "older people"[Title/Abstract] OR "old person"[Title/Abstract] OR "older person"[Title/Abstract] OR "older communit*"[Title/Abstract] OR "older population*"[Title/Abstract] OR "senior people"[Title/Abstract] OR "senior person"[Title/Abstract] OR "senior citizen*"[Title/Abstract] OR "senior communit*"[Title/Abstract] OR "senior population*"[Title/Abstract] OR geriatric*[Title/Abstract] OR "oldest old"[Title/Abstract] OR "old age"[Title/Abstract] OR "old aged"[Title/Abstract] OR "older age"[Title/Abstract] OR "older aged"[Title/Abstract] OR "over 65"[Title/Abstract] OR "over 60"[Title/Abstract] OR "over 70"[Title/Abstract] OR "over 75"[Title/Abstract] OR "over 80"[Title/Abstract] OR "over 85"[Title/Abstract] OR "over 90"[Title/Abstract] OR "over 95"[Title/Abstract] OR "over 6*"[Title/Abstract] OR "over 7*"[Title/Abstract] OR "over 8*"[Title/Abstract] OR "over 9*"[Title/Abstract] OR aged[MeSH Terms])  AND  (Sleep[Title/Abstract] OR "sleep pattern"[Title/Abstract] OR "sleep quality"[Title/Abstract] OR "sleep duration"[Title/Abstract] OR "sleep continuity"[Title/Abstract] OR "sleep fragmentation"[Title/Abstract] OR "sleep architecture"[Title/Abstract] OR "sleep disturbance"[Title/Abstract] OR "sleep disorder"[Title/Abstract] OR insomnia[Title/Abstract] OR "sleep deprivation"[Title/Abstract] OR "sleep apnea"[Title/Abstract] OR "restless legs syndrome"[Title/Abstract] OR RLS[Title/Abstract] OR narcolepsy[Title/Abstract] OR parasomnia[Title/Abstract] OR "circadian rhythm disorders"[Title/Abstract] OR hypersomnia[Title/Abstract] OR "sleep-wake disorders"[Title/Abstract] OR sleep[MeSH Terms] OR sleep wake disorders[MeSH Terms])  AND  (balance[Title/Abstract] OR "static balance"[Title/Abstract] OR "dynamic balance"[Title/Abstract] OR "postural control"[Title/Abstract] OR "posture equilibriums"[Title/Abstract] OR "posture balance"[Title/Abstract] OR "posture control"[Title/Abstract] OR postural balance[MeSH Terms] OR core stability[MeSH Terms])  AND  (cognition[Title/Abstract] OR "cognitive*"[Title/Abstract] OR "cognitive ability"[Title/Abstract] OR "cognitive function"[Title/Abstract] OR memory[Title/Abstract] OR "episodic memory"[Title/Abstract] OR "declarative memory"[Title/Abstract] OR "short-term memory"[Title/Abstract] OR "long-term memory"[Title/Abstract] OR "working memory"[Title/Abstract] OR "verbal memory"[Title/Abstract] OR "spatial memory"[Title/Abstract] OR "object memory"[Title/Abstract] OR "location memory"[Title/Abstract] OR encoding[Title/Abstract] OR storage[Title/Abstract] OR retrieval[Title/Abstract] OR "free recall"[Title/Abstract] OR "cued recall"[Title/Abstract] OR "forced-choice recognition"[Title/Abstract] OR recall[Title/Abstract] OR "procedural memory"[Title/Abstract] OR "semantic memory"[Title/Abstract] OR "prospective memory"[Title/Abstract] OR "executive function"[Title/Abstract] OR "executive functioning"[Title/Abstract] OR reasoning[Title/Abstract] OR "problem-solving"[Title/Abstract] OR processing[Title/Abstract] OR "processing speed"[Title/Abstract] OR fluency[Title/Abstract] OR coding[Title/Abstract] OR tracking[Title/Abstract] OR execution[Title/Abstract] OR executive[Title/Abstract] OR maintenance[Title/Abstract] OR manipulation[Title/Abstract] OR attention[Title/Abstract] OR concentration[Title/Abstract] OR selective attention[Title/Abstract] OR "sustained attention"[Title/Abstract] OR vigilance[Title/Abstract] OR multisensory[Title/Abstract] OR perception[Title/Abstract] OR "object recognition"[Title/Abstract] OR "organizational strategies"[Title/Abstract] OR naming[Title/Abstract] OR reading[Title/Abstract] OR comprehension[Title/Abstract] OR mental[Title/Abstract] OR arousal[Title/Abstract] OR cognition[MeSH Terms] OR Cognition Disorders[MeSH Terms] OR Mental Processes[MeSH Terms])  Filters: from 2004 - 2024  Total: 188 (March 1, 2024)   - Web of Science   (TS=("older adult*" OR "old adult*" OR elder* OR "old people" OR "older people" OR "old person" OR "older person" OR "older communit*" OR "older population*" OR "senior people" OR "senior person" OR "senior citizen*" OR "senior communit*" OR "senior population*" OR geriatric* OR "oldest old" OR "old age" OR "old aged" OR "older age" OR "older aged" OR "over 65" OR "over 60" OR "over 70" OR "over 75" OR "over 80" OR "over 85" OR "over 90" OR "over 95" OR "over 6*" OR "over 7*" OR "over 8*" OR "over 9*"))  AND  (TS=(Sleep OR “sleep pattern” OR “sleep quality” OR “sleep duration” OR “sleep continuity” OR “sleep fragmentation” OR “sleep architecture” OR “sleep disturbance” OR “sleep disorder” OR insomnia OR “sleep deprivation” OR “sleep apnea” OR “restless legs syndrome” OR RLS OR narcolepsy OR parasomnia OR “circadian rhythm disorders” OR hypersomnia OR “sleep-wake disorders”))  AND  (TS=(cognition OR “cognitive*” OR “cognitive ability” OR “cognitive function” OR memory OR “episodic memory” OR “declarative memory” OR “short-term memory” OR “long-term memory” OR “working memory” OR “verbal memory” OR “spatial memory” OR “object memory” OR “location memory” OR encoding OR storage OR retrieval OR “free recall” OR “cued recall” OR “forced-choice recognition” OR recall OR “procedural memory” OR “semantic memory” OR “prospective memory” OR “executive function” OR “executive functioning” OR reasoning OR “problem-solving” OR processing OR “processing speed” OR fluency OR coding OR tracking OR execution OR executive OR maintenance OR manipulation OR attention OR concentration OR selective attention OR “sustained attention” OR vigilance OR multisensory OR perception OR “object recognition” OR “organizational strategies” OR naming OR reading OR comprehension OR mental OR arousal))  AND  (TS=(balance OR “static balance” OR “dynamic balance” OR “postural control” OR “posture equilibriums” OR “posture balance” OR “posture control”))  Refined by: Publication years: 2004 ~ 2024  Total: 252 (March 1, 2024)   - CINHAIL   S1 AND S2 AND S3 AND S4  S1:  (MH "Aged+") OR TI ( "older adult*" OR "old adult*" OR elder* OR "old people" OR "older people" OR "old person" OR "older person" OR "older communit*" OR "older population*" OR "senior people" OR "senior person" OR "senior citizen*" OR "senior communit*" OR "senior population*" OR geriatric* OR "oldest old" OR "old age" OR "old aged" OR "older age" OR "older aged" OR "over 65" OR "over 60" OR "over 70" OR "over 75" OR "over 80" OR "over 85" OR "over 90" OR "over 95" OR "over 6*" OR "over 7*" OR "over 8*" OR "over 9*" ) OR AB ( "older adult*" OR "old adult*" OR elder* OR "old people" OR "older people" OR "old person" OR "older person" OR "older communit*" OR "older population*" OR "senior people" OR "senior person" OR "senior citizen*" OR "senior communit*" OR "senior population*" OR geriatric* OR "oldest old" OR "old age" OR "old aged" OR "older age" OR "older aged" OR "over 65" OR "over 60" OR "over 70" OR "over 75" OR "over 80" OR "over 85" OR "over 90" OR "over 95" OR "over 6*" OR "over 7*" OR "over 8*" OR "over 9*" )  S2:  ( (MH "Sleep Pattern Disturbance (Saba CCC)") OR (MH "Sleep Pattern Disturbance (NANDA)") OR (MH "Sleep Disorders, Circadian Rhythm+") OR (MH "Sleep Apnea, Central+") OR (MH "Sleep Apnea, Obstructive") OR (MH "Sleep Disorders+") OR (MH "Sleep-Wake Transition Disorders+") OR (MH "Sleep Disorders, Intrinsic+") OR (MH "Sleep Apnea Syndromes+") OR (MH "Parasomnias+") OR (MH "Deep Sleep") OR (MH "Sleep Arousal Disorders+") OR (MH "Sleep+") ) OR TI ( Sleep OR “sleep pattern” OR “sleep quality” OR “sleep duration” OR “sleep continuity” OR “sleep fragmentation” OR “sleep architecture” OR “sleep disturbance” OR “sleep disorder” OR insomnia OR “sleep deprivation” OR “sleep apnea” OR “restless legs syndrome” OR RLS OR narcolepsy OR parasomnia OR “circadian rhythm disorders” OR hypersomnia OR “sleep-wake disorders” ) OR AB ( Sleep OR “sleep pattern” OR “sleep quality” OR “sleep duration” OR “sleep continuity” OR “sleep fragmentation” OR “sleep architecture” OR “sleep disturbance” OR “sleep disorder” OR insomnia OR “sleep deprivation” OR “sleep apnea” OR “restless legs syndrome” OR RLS OR narcolepsy OR parasomnia OR “circadian rhythm disorders” OR hypersomnia OR “sleep-wake disorders” )  S3:  ( (MH "Balance, Postural+") OR (MH "Core Stability") ) OR TI ( balance OR “static balance” OR “dynamic balance” OR “postural control” OR “posture equilibriums” OR “posture balance” OR “posture control” ) OR AB ( balance OR “static balance” OR “dynamic balance” OR “postural control” OR “posture equilibriums” OR “posture balance” OR “posture control” )  S4:  ( (MH "Cognition+") OR (MH "Cognition Disorders+") OR (MH "Cognitive Therapy+") OR (MH "Delirium, Dementia, Amnestic, Cognitive Disorders+") OR (MH "Mental Processes+") OR (MH "Mental Disorders+") ) OR TI ( cognition OR “cognitive*” OR “cognitive ability” OR “cognitive function” OR memory OR “episodic memory” OR “declarative memory” OR “short-term memory” OR “long-term memory” OR “working memory” OR “verbal memory” OR “spatial memory” OR “object memory” OR “location memory” OR encoding OR storage OR retrieval OR “free recall” OR “cued recall” OR “forced-choice recognition” OR recall OR “procedural memory” OR “semantic memory” OR “prospective memory” OR “executive function” OR “executive functioning” OR reasoning OR “problem-solving” OR processing OR “processing speed” OR fluency OR coding OR tracking OR execution OR executive OR maintenance OR manipulation OR attention OR concentration OR selective attention OR “sustained attention” OR vigilance OR multisensory OR perception OR “object recognition” OR “organizational strategies” OR naming OR reading OR comprehension OR mental OR arousal ) OR AB ( cognition OR “cognitive*” OR “cognitive ability” OR “cognitive function” OR memory OR “episodic memory” OR “declarative memory” OR “short-term memory” OR “long-term memory” OR “working memory” OR “verbal memory” OR “spatial memory” OR “object memory” OR “location memory” OR encoding OR storage OR retrieval OR “free recall” OR “cued recall” OR “forced-choice recognition” OR recall OR “procedural memory” OR “semantic memory” OR “prospective memory” OR “executive function” OR “executive functioning” OR reasoning OR “problem-solving” OR processing OR “processing speed” OR fluency OR coding OR tracking OR execution OR executive OR maintenance OR manipulation OR attention OR concentration OR selective attention OR “sustained attention” OR vigilance OR multisensory OR perception OR “object recognition” OR “organizational strategies” OR naming OR reading OR comprehension OR mental OR arousal)  Limiters - Publication Date: 20040101-20241231  Total: 120 (March 1, 2024)   - Embase   ('old adult*':ab,ti OR elder*:ab,ti OR 'old people':ab,ti OR 'older people':ab,ti OR 'old person':ab,ti OR 'older person':ab,ti OR 'older communit*':ab,ti OR 'older population*':ab,ti OR 'senior people':ab,ti OR 'senior person':ab,ti OR 'senior citizen*':ab,ti OR 'senior communit*':ab,ti OR 'senior population*':ab,ti OR geriatric*:ab,ti OR 'oldest old':ab,ti OR 'old age':ab,ti OR 'old aged':ab,ti OR 'older age':ab,ti OR 'older aged':ab,ti OR 'over 65':ab,ti OR 'over 60':ab,ti OR 'over 70':ab,ti OR 'over 75':ab,ti OR 'over 80':ab,ti OR 'over 85':ab,ti OR 'over 90':ab,ti OR 'over 95':ab,ti OR 'over 6*':ab,ti OR 'over 7*':ab,ti OR 'over 8*':ab,ti OR 'over 9*':ab,ti OR 'aged'/exp OR 'very elderly'/exp)  AND  (sleep:ab,ti OR 'sleep pattern':ab,ti OR 'sleep quality':ab,ti OR 'sleep duration':ab,ti OR 'sleep continuity':ab,ti OR 'sleep fragmentation':ab,ti OR 'sleep architecture':ab,ti OR 'sleep disturbance':ab,ti OR 'sleep disorder':ab,ti OR insomnia:ab,ti OR 'sleep deprivation':ab,ti OR 'sleep apnea':ab,ti OR 'restless legs syndrome':ab,ti OR rls:ab,ti OR narcolepsy:ab,ti OR parasomnia:ab,ti OR 'circadian rhythm disorders':ab,ti OR hypersomnia:ab,ti OR 'sleep-wake disorders':ab,ti OR 'sleep'/exp OR 'sleep disorder'/exp)  AND  (cognition:ab,ti OR 'cognitive*':ab,ti OR 'cognitive ability':ab,ti OR 'cognitive function':ab,ti OR memory:ab,ti OR 'episodic memory':ab,ti OR 'declarative memory':ab,ti OR 'short-term memory':ab,ti OR 'long-term memory':ab,ti OR 'working memory':ab,ti OR 'verbal memory':ab,ti OR 'spatial memory':ab,ti OR 'object memory':ab,ti OR 'location memory':ab,ti OR encoding:ab,ti OR storage:ab,ti OR retrieval:ab,ti OR 'free recall':ab,ti OR 'cued recall':ab,ti OR 'forced-choice recognition':ab,ti OR recall:ab,ti OR 'procedural memory':ab,ti OR 'semantic memory':ab,ti OR 'prospective memory':ab,ti OR 'executive function':ab,ti OR 'executive functioning':ab,ti OR reasoning:ab,ti OR 'problem-solving':ab,ti OR processing:ab,ti OR 'processing speed':ab,ti OR fluency:ab,ti OR coding:ab,ti OR tracking:ab,ti OR execution:ab,ti OR executive:ab,ti OR maintenance:ab,ti OR manipulation:ab,ti OR attention:ab,ti OR concentration:ab,ti OR 'selective attention':ab,ti OR 'sustained attention':ab,ti OR vigilance:ab,ti OR multisensory:ab,ti OR perception:ab,ti OR 'object recognition':ab,ti OR 'organizational strategies':ab,ti OR naming:ab,ti OR reading:ab,ti OR comprehension:ab,ti OR mental:ab,ti OR arousal:ab,ti OR 'cognition'/exp OR 'cognitive defect'/exp OR 'mental function'/exp)  AND  (balance:ab,ti OR 'static balance':ab,ti OR 'dynamic balance':ab,ti OR 'postural control':ab,ti OR 'posture equilibriums':ab,ti OR 'posture balance':ab,ti OR 'posture control':ab,ti OR 'body equilibrium'/exp OR 'core stability'/exp)  Filtered: Publication years 2004 ~2024  Total: 794 (March 1, 2024) |

Table A2. Eligibility Criteria

| Title/Abstract Screening Criteria | |
| --- | --- |
| Inclusion criteria | - The outcome assessment or intervention must include at least two target components: sleep, cognitive function, and balance (including static, dynamic, and postural balance).   - Although the target components were not mentioned in the outcome assessment, if used in the subgroup analysis, they will be considered to have the target components. - Human target research |
| Exclusion criteria | - Two of the three target components are included in the intervention or outcome assessment, but they will not be counted if   - A multi-component intervention includes 2 or more components not related to the target components or   - 2) The target components are not reported as key intervention components in a multi-component intervention     - (e.g., educational sessions on diet and sleep included in 10 weeks of supervised moderate-intensity aerobic and resistance training). - Interested components are not mentioned as key intervention or outcome assessment components.   - (e.g., Side effect of the medication or characteristic of certain disease). - The type of the study was conference abstracts, protocols, book reviews, or editorials. - The type of the study was a literature review, research protocol, or intervention or assessment development/validation study.   - Literature review papers will be included for additional search only if they have the same criteria for literature collection in the target population and three interested components.   - Research protocol will be included for additional search for further study only if the target population and components meet the screening criteria. - Reported that the target population includes those under 60 years old. - Balance in the study is not postural balance or core stability, for example, hormone balance, nutrition balance, or chemical balance. - Not written in English |
| Full-Text Review Criteria | |
| Inclusion criteria | - The outcome assessment or intervention must include at least three target components which are sleep, cognitive function, postural balance, and related components of each (fall is different from the balance).   - Although the target components were not mentioned in the outcome assessment if used in the subgroup analysis, they will be considered to have the target components. - Relationships between interested components must be analyzed or described.   - Possible scenarios (interested components A, B, and C) (these scenarios do not cover all the possible scenarios)     - The study analyzed the correlation between A, B, and C.       - e.g., the multivariate relationship between sleep duration, postural balance, and processing speed was analyzed.     - The study analyzed the correlation between A and B and did the subgroup analysis based on the level of C.       - e.g., the correlation between sleep duration and postural balance was analyzed in each subgroup of dementia, mild cognitive decline, and no cognitive decline.     - The study examined the correlation between A and B, with C acting as a confounding or contributing factor.       - e.g., the relationship between sleep duration and postural balance is changed by controlling for overall cognitive function. - Human target research |
| Exclusion criteria | - Three target components are included in the intervention or outcome assessment, but they will not be counted if   - 1) A multi-component intervention includes 2 or more components not related to the target components or   - 2) The target components are not reported as key intervention components in a multi-component intervention     - (e.g., educational sessions on diet and sleep included in 10 weeks of supervised moderate-intensity aerobic and resistance training).   - 3) The balance in the study is not postural balance or core stability, for example, hormone balance, nutrition balance, or chemical balance. - Interested components are included in the study, but the relationship or effect is not analyzed or mentioned.   - (e.g., Side effect of the medication or characteristic of a certain disease). - The type of study was conference abstracts, protocols, book reviews, or editorials. - The type of study was a literature review, research protocol, intervention, or assessment development/validation study.   - Literature review papers will be included for additional search only if they have the same criteria for literature collection in the target population and three interested components.   - Research protocol will be included for additional search for further study only if the target population and components are meeting with the screening criteria. - Reported that the target population includes those under 60 years old. - Not written in English - Cannot access the full-text - No full-text |

Table A3. Description of included observational studies

| First author  Published year  Country | Study design  Level of evidence (LoE)  Study type | Target population  Subgroups | Sample size (baseline / analyzed) |
| --- | --- | --- | --- |
| Alasmari [(1)](https://sciwheel.com/work/citation?ids=16606276&pre=&suf=&sa=0&dbf=0)  2023  Saudi Arabia | Cross-Sectional Study  LoE: IV  A | Older adults with health conditions (diabetes) | 309/309 |
| Amjad [(2)](https://sciwheel.com/work/citation?ids=8574923&pre=&suf=&sa=0&dbf=0)  2019  USA | Cross-Sectional Study  LoE: IV  B | Community-dwelling older adults | 1270/1270 |
| Arena [(3)](https://sciwheel.com/work/citation?ids=2984774&pre=&suf=&sa=0&dbf=0)  2016  USA | Prospective cohort design  LoE: II  A | Older adults with health conditions (progressive supranuclear palsy)  No subgroups | 35/35 |
| Catikkas [(4)](https://sciwheel.com/work/citation?ids=16590956&pre=&suf=&sa=0&dbf=0)  2023  Turkey | Cross-Sectional Study  LoE: IV  C | Older adults with health conditions (diabetes) | 227/227 |
| Chen [(5)](https://sciwheel.com/work/citation?ids=16590948&pre=&suf=&sa=0&dbf=0)  2020  Taiwan | Prospective Cohort Study  LoE: II  B | Older adults with health conditions (MCI) | 100/74 |
| Dokuzlar [(6)](https://sciwheel.com/work/citation?ids=7731911&pre=&suf=&sa=0&dbf=0) 2017  Turkey | Cross-Sectional Study  LoE: IV  A | Older adults (who presented at the geriatric polyclinics of a university hospital) | 335/335 |
| Engin [(7)](https://sciwheel.com/work/citation?ids=8576076&pre=&suf=&sa=0&dbf=0)  2010  Turkey | Cross-Sectional Study  LoE: IV  A | Community-dwelling older adults (who were registered to a community health service home care; IMM) | 1163/1163 |
| Ganidagli [(8)](https://sciwheel.com/work/citation?ids=15671536&pre=&suf=&sa=0&dbf=0)  2023  Turkey | Cross-Sectional Study  LoE: IV  B | Older adults (who were attending the geriatric outpatient clinic) | 237/237 |
| Gazzola [(9)](https://sciwheel.com/work/citation?ids=12411209&pre=&suf=&sa=0&dbf=0)  2009  Brazil | Cross-Sectional Study  LoE: IV  A | Older adults with health conditions (chronic vestibular dysfunction) | 120/120 |
| Lichter [(10)](https://sciwheel.com/work/citation?ids=14104016&pre=&suf=&sa=0&dbf=0)  2021  USA | Cross-sectional study (retrospective)  LoE: IV  A | Older adults with health conditions (Parkinson’s disease) | 164/164 |
| Louis [(11)](https://sciwheel.com/work/citation?ids=16590942&pre=&suf=&sa=0&dbf=0)  2016  USA | Prospective Observational Study  LoE: IV  A | Older adults with health conditions (essential tremor (ET) or Parkinson's disease (PD) (ET-PD patients) or both) | 171/171 |
| Nisser [(12)](https://sciwheel.com/work/citation?ids=16590959&pre=&suf=&sa=0&dbf=0)  2022  Germany | Cross-Sectional Study  LoE: IV  B | Older adults with health conditions (clinically isolated REM sleep behaviour disorder) | 40/40 |
| Oytun [(13)](https://sciwheel.com/work/citation?ids=16606280&pre=&suf=&sa=0&dbf=0)  2023  Turkey | Cross-Sectional Study  LoE: IV  A | Older adults with health conditions (probable mild-to-moderate Alzheimer’s disease) | 56/56 |
| Pahwa [(14)](https://sciwheel.com/work/citation?ids=8573920&pre=&suf=&sa=0&dbf=0)  2016  USA | Cross-Sectional Study  LoE: IV  A | Older adults with health conditions (community-dwelling women with urinary incontinence (UI) | 37/37 |
| Paker [(15)](https://sciwheel.com/work/citation?ids=16590955&pre=&suf=&sa=0&dbf=0)  2018  Turkey | Cross-Sectional Study  LoE: IV  B | Community-dwelling older adults (attending outpatient clinics) | 160/160 |
| Rosenberg [(16)](https://sciwheel.com/work/citation?ids=1499793&pre=&suf=&sa=0&dbf=0)  2016  USA | Cross-Sectional Study  LoE: IV  A | Community-dwelling older adults (retirement communities) | 307/307 |
| Scharre [(17)](https://sciwheel.com/work/citation?ids=3296761&pre=&suf=&sa=0&dbf=0)  2016  USA | Cross-Sectional Study  LoE: IV  B | Older adults with health conditions (Lewy body dementia, Alzheimer's disease, or Parkinson's disease) | 72/63 |
| Schnittger [(18)](https://sciwheel.com/work/citation?ids=8574132&pre=&suf=&sa=0&dbf=0)  2012  Ireland | Cross-Sectional Study  LoE: IV  B | Community-dwelling older adults | 579/579 |
| Schrag [(19)](https://sciwheel.com/work/citation?ids=2407123&pre=&suf=&sa=0&dbf=0)  2015  United Kingdom | Case-Control Study  LoE: III-2  A | Older adults with health conditions (with first diagnosis of Parkinson’s disease and those without Parkinson’s disease) | 54921/54921 |
| Si [(20)](https://sciwheel.com/work/citation?ids=8575013&pre=&suf=&sa=0&dbf=0)  2019  China | Cross-Sectional Study  LoE: IV  A | Community-dwelling older adults | 541/541 |
| Soysal [(21)](https://sciwheel.com/work/citation?ids=14299967&pre=&suf=&sa=0&dbf=0)  2023  Turkey | Cross-Sectional Study  LoE: IV  A | Older adults with health conditions (Lewy body dementia or Alzheimer's disease) | 350/350 |
| Swiatkowska [(22)](https://sciwheel.com/work/citation?ids=9689507&pre=&suf=&sa=0&dbf=0)  2022  England | Case-Control Study  LoE: III-2  A | Older adults with health conditions (patients With Highly Elevated Blood Cobalt) | 106/106 |
| Wang [(23)](https://sciwheel.com/work/citation?ids=13263451&pre=&suf=&sa=0&dbf=0)  2022  China | Cross-Sectional Study  LoE: IV  A | Community-dwelling older adults | 669/669 |
| Yamada [(24)](https://sciwheel.com/work/citation?ids=8574373&pre=&suf=&sa=0&dbf=0)  2014  European Countries | Cross-Sectional Study  LoE: IV  A | Nursing home residents | 4007/4007 |
| Zahirovic [(25)](https://sciwheel.com/work/citation?ids=16590981&pre=&suf=&sa=0&dbf=0)  2019  Sweden | Prospective Cohort Study  LoE: II  A | Nursing home residents (whose core LBD signs, electronic hospital records, and electronic medication lists were available) | 583/583 |
| Zhang [(26)](https://sciwheel.com/work/citation?ids=16590949&pre=&suf=&sa=0&dbf=0)  2023  China | Cross-Sectional Study  LoE: IV  A | Community-dwelling older adults | 541/541 |

Table A4. Description of included experimental studies

| First author  Published year  Country | Study design  Level of evidence (LoE)  Study type | Target population  Subgroups | Sample size (baseline / analyzed) |
| --- | --- | --- | --- |
| Uemura [(27)](https://sciwheel.com/work/citation?ids=16606288&pre=&suf=&sa=0&dbf=0)  2015  Japan | RCT (4-period)  LoE: II  A | Healthy older adults | 13/13 |
| Yoon [(28)](https://sciwheel.com/work/citation?ids=16590947&pre=&suf=&sa=0&dbf=0)  2019  South Korea | RCT  LoE: II  A | Older women (who were physically healthy and enrolled in the National Fitness Center in Seoul | 30/30 |
| Vogel [(29)](https://sciwheel.com/work/citation?ids=13170619&pre=&suf=&sa=0&dbf=0) 2021  Germany | RCT  LoE: II  B | Community-dwelling older adults | 49/37 |
| Kamoun [(30)](https://sciwheel.com/work/citation?ids=16590940&pre=&suf=&sa=0&dbf=0)  2024  Tunisia | RCT  LoE: II  A | Older adults (who were physically active) | 28/20 |
| Tsunoda [(31)](https://sciwheel.com/work/citation?ids=2357295&pre=&suf=&sa=0&dbf=0)  2010  Japan | Quasi-experimental study (one-group pretest–posttest)  LoE: IV  A | Nursing home residents who were receiving Benzodiazepine (BZD) | 30/30 |
| Baek [(32)](https://sciwheel.com/work/citation?ids=16590939&pre=&suf=&sa=0&dbf=0)  2022  South Korea | Quasi-experimental study (one-group pretest–posttest)  LoE: IV  A | Older adults with health conditions (MCI) | 23/22 |
| Syed-Abdul [(33)](https://sciwheel.com/work/citation?ids=16606292&pre=&suf=&sa=0&dbf=0)  2022  USA | Quasi-experimental study (one-group pretest–posttest)  LoE: IV  A | Older adults (who were registered in the strength training program, SSSH) | 20/20 |

Table A5. Instruments and methods used to measure the sleep domains

| Sleep components | Measures used |
| --- | --- |
| Sleep Quality | - Pittsburgh Sleep Quality Index (PSQI) - Self-designed questionnaire or interview - Leeds Sleep Evaluation Questionnaire (LSEQ) - Spiegel sleep questionnaire |
| Prevalence or incidence of sleep-related conditions | - The Neurotoxic Symptom Checklist-60 (NSC-60) (sleep disturbance) - Electronic health records (insomnia, REM sleep behavior disorder) - 6-item short forms from the Patient-Reported Outcomes Measurement Information System (pain interference and sleep disturbance) - Swedish National Medication Dispensing System (rapid eye movement sleep behavior disorder) - Comprehensive geriatric assessment (Daytime sleepiness) - International Classification of Sleep Disorders (isolated REM sleep behavior disorder) - Phasic electromyography (Atonia) - Mayo Sleep Questionnaire-Informant (REM sleep behavior disorder) - Self-designed questionnaire or interview (report on various sleep problems: |
| Severity of sleep-related conditions | - Epworth Sleepiness Scale - Insomnia Severity Index - MDS-UPDRS nonmotor (nM) symptoms questionnaire - Stanford sleepiness scale |

Table A6. Instruments and methods used to measure the balance domains

| Balance components | Measures used |
| --- | --- |
| Overall Balance | - Short physical performance battery - Tinetti Performance Oriented Mobility Assessment - Berg Balance Scale - Mini-Berg test |
| Static Balance | - Clinical stabilometric or force platform system - Chair stand test - Sit and reach test - One-leg stance test |
| Dynamic Balance | - 8-feet up and go test - Functional reach test - Timed up and go test - Dynamic Gait Index - Straight Line Walk Test - Timed 25-feet walk test |
| Balance Confidence | - Activities-specific balance confidence scale |
| Prevalence or incidence of balance-related conditions | - The Neurotoxic Symptom Checklist-60 (balance disturbance) - Electronic health records (balance impairments) - Swedish National Medication Dispensing System - Self-designed questionnaire or interview (difficulties or impairments in balance) |
| Severity of balance-related conditions | - Postural instability and gait difficulty in Unified Parkinson's Disease Rating Scale |

Table A7. Instruments and methods used to measure the cognition domains

| Cognition components | Measures used |
| --- | --- |
| Global Cognition | - Mini-mental state examination - Repeatable Battery for the Assessment of Neuropsychological Status - Montreal Cognitive Assessment - Cognitive state test - Cognitive Performance Scale - Mini-Cog test - Self-administered gerocognitive examination - Consortium to establish a registry for Alzheimer’s disease - Alzheimer’s Disease Assessment Scale - Clinical dementia rating - Frontal assessment battery   Korean dementia screening questionnaire-cognition |
| Executive function | - Trail-making test A - Trail-making test B - Wisconsin Card Sort Task-64 |
| Memory | - Subjective memory complaint questionnaire - Auditory verbal learning test - Forward digit span - California verbal language test-II - Digit recall forward and backward - Taylor Complex Figure Test - Short-term memory test |
| Visual-spatial function | - Clock drawing test - Benton Judgment of Line Orientation |
| Verbal fluency | - Verbal fluency test - FAS verbal fluency test |
| Confrontation naming ability | - Modified (15-word) Boston naming test |
| Semantic fluency | - Animal naming test |
| Structural language skills | - Cookie theft picture |
| Patient-reported clinical symptoms of cognition | - The Neurotoxic Symptom Checklist-60 (cognitive defect) - Electronic health records (memory and cognitive decline) - Swedish National Medication Dispensing System (dementia diagnosis and fluctuating cognition) - DSM-IV guidelines (dementia) - Nonmotor symptoms questionnaire in unified Parkinson’s disease rating scale - Self-designed questionnaire or interview (neurological and/or cognitive impairments) |

Table A8. Summary of psychometric properties and constructs of assessment tools

| Tool Name | Domain | Construct and type | Psychometric Properties | Ref |
| --- | --- | --- | --- | --- |
| Pittsburgh Sleep Quality Index (PSQI) | Sleep | Subjective sleep quality  Subjective, self-report | Internal consistency: Cronbach’s α=0.69  Construct validity: Supported by correlations with objective sleep measure | [(34)](https://sciwheel.com/work/citation?ids=2705811&pre=&suf=&sa=0&dbf=0) |
| Timed Up and Go (TUG) | Balance | Dynamic balance  Objective, performance-based | Interrater and test-retest reliability: ICC=0.98 and 0.50  Predictive of falls risk: >13.5 s cutoff for 87% sensitivity | [(35,36)](https://sciwheel.com/work/citation?ids=2156155,5929751&pre=&pre=&suf=&suf=&sa=0,0&dbf=0&dbf=0) |
| Berg Balance Scale (BBS) | Balance | Overall balance  Objective, performance-based | Internal consistency: Cronbach’s α=0.96  Interrater and test-retest reliability: ICC=0.98 and 0.97 | [(37,38)](https://sciwheel.com/work/citation?ids=2043839,5392304&pre=&pre=&suf=&suf=&sa=0,0&dbf=0&dbf=0) |
| Tinetti Performance-Oriented Mobility Assessment (POMA) | Balance | Overall balance  Objective, performance-based | Interrater and test-retest reliability: ICC=0.94-0.97 and 0.84/0.96-0.97  Predictive validity of falls: 70-85% | [(39–41)](https://sciwheel.com/work/citation?ids=11294273,5373815,5753811&pre=&pre=&pre=&suf=&suf=&suf=&sa=0,0,0&dbf=0&dbf=0&dbf=0) |
| Activities-specific Balance Confidence (ABC) Scale | Balance | Balance confidence  Subjective, self-report | Internal consistency: Cronbach’s α=0.96  Test-retest reliability: r=0.92, *p*<0.001 | [(37,42)](https://sciwheel.com/work/citation?ids=2043839,2097247&pre=&pre=&suf=&suf=&sa=0,0&dbf=0&dbf=0) |
| Mini-Mental State Examination (MMSE) | Cognition | Global cognition  Objective, widely used screening tool | Internal consistency: Cronbach’s α=0.78  Test-retest reliability: r=0.56-0.80 | [(43,44)](https://sciwheel.com/work/citation?ids=10112654,17921165&pre=&pre=&suf=&suf=&sa=0,0&dbf=0&dbf=0) |
| Consortium to Establish a Registry for Alzheimer's Disease (CERAD) | Cognition | Global cognition  Objective, comprehensive battery | Interrater and test-retest reliability: r=0.87 and 0.77, *p*<0.001 | [(45)](https://sciwheel.com/work/citation?ids=2405022&pre=&suf=&sa=0&dbf=0) |
| Unified Parkinson's Disease Rating Scale Part III (UPDRS III) | Balance | Overall balance (PD-specific)  Objective, clinician-administered | Internal consistency: Cronbach’s α=0.96  Test-retest reliability: ICC=0.93 | [(46)](https://sciwheel.com/work/citation?ids=8915200&pre=&suf=&sa=0&dbf=0) |
| Self-Administered Gerocognitive Examination (SAGE) | Cognition | Global cognition, dementia screening  Objective, screening tool | Correlation with MMSE: r=0.76  Interrater and test-retest reliability: ICC=0.96 | [(47)](https://sciwheel.com/work/citation?ids=5845782&pre=&suf=&sa=0&dbf=0) |
| Montreal Cognitive Assessment (MoCA) | Cognition | Global cognition  Objective, widely used screening tool | Internal consistency: Cronbach’s α=0.83  Test-retest reliability: ICC=0.92 | [(48)](https://sciwheel.com/work/citation?ids=1028118&pre=&suf=&sa=0&dbf=0) |
| Functional Reach Test (FRT) | Balance | Static balance  Objective, performance-based | Test-retest reliability: ICC=0.83 | [(49)](https://sciwheel.com/work/citation?ids=15171828&pre=&suf=&sa=0&dbf=0) |
| Straight Line Walking Test (SLWT) | Balance | Dynamic balance  Objective, performance-based | Interrater and test-retest reliability: ICC=0.88-0.96 and 0.86-0.91 | [(50)](https://sciwheel.com/work/citation?ids=14897564&pre=&suf=&sa=0&dbf=0) |

Table A9. Risk of bias in the included observational studies (n=26)

| First author and year | Study design | Selection | Comparability | Outcome | Total score |
| --- | --- | --- | --- | --- | --- |
| Alasmari [(1)](https://sciwheel.com/work/citation?ids=16606276&pre=&suf=&sa=0&dbf=0)  2023 | Cross-Sectional Study | ★★★★☆ | ★★ | ★★☆ | 8/10 |
| Amjad [(2)](https://sciwheel.com/work/citation?ids=8574923&pre=&suf=&sa=0&dbf=0)  2019 | Cross-Sectional Study | ★★★★☆ | ★★ | ★★☆ | 8/10 |
| Arena [(3)](https://sciwheel.com/work/citation?ids=2984774&pre=&suf=&sa=0&dbf=0)  2016 | Prospective Cohort Study | ★★★☆ | ★☆ | ★★★ | 7/9 |
| Catikkas [(4)](https://sciwheel.com/work/citation?ids=16590956&pre=&suf=&sa=0&dbf=0)  2023 | Cross-Sectional Study | ★★★★☆ | ★★ | ★★☆ | 8/10 |
| Chen [(5)](https://sciwheel.com/work/citation?ids=16590948&pre=&suf=&sa=0&dbf=0)  2020 | Prospective Cohort Study | ★★★☆ | ★☆ | ★★★ | 7/9 |
| Dokuzlar [(6)](https://sciwheel.com/work/citation?ids=7731911&pre=&suf=&sa=0&dbf=0) 2017 | Cross-Sectional Study | ★★★★☆ | ★☆ | ★★☆ | 7/10 |
| Engin [(7)](https://sciwheel.com/work/citation?ids=8576076&pre=&suf=&sa=0&dbf=0)  2010 | Cross-Sectional Study | ★★★★☆ | ★★ | ★★☆ | 8/10 |
| Ganidagli [(8)](https://sciwheel.com/work/citation?ids=15671536&pre=&suf=&sa=0&dbf=0)  2023 | Cross-Sectional Study | ★★★☆☆ | ★★ | ★★☆ | 7/10 |
| Gazzola [(9)](https://sciwheel.com/work/citation?ids=12411209&pre=&suf=&sa=0&dbf=0)  2009 | Cross-Sectional Study | ★★★☆☆ | ★★ | ★★☆ | 7/10 |
| Lichter [(10)](https://sciwheel.com/work/citation?ids=14104016&pre=&suf=&sa=0&dbf=0)  2021 | Cross-sectional study (retrospective) | ★★★★☆ | ★★ | ★★☆ | 8/10 |
| Louis [(11)](https://sciwheel.com/work/citation?ids=16590942&pre=&suf=&sa=0&dbf=0)  2016 | Prospective Observational Study (cross-sectional analysis) | ★★★★☆ | ★★ | ★★★ | 9/10 |
| Nisser [(12)](https://sciwheel.com/work/citation?ids=16590959&pre=&suf=&sa=0&dbf=0)  2022 | Cross-Sectional Study | ★★★☆☆ | ★★ | ★★☆ | 7/10 |
| Oytun [(13)](https://sciwheel.com/work/citation?ids=16606280&pre=&suf=&sa=0&dbf=0)  2023 | Cross-Sectional Study | ★★★☆☆ | ★★ | ★★☆ | 7/10 |
| Pahwa [(14)](https://sciwheel.com/work/citation?ids=8573920&pre=&suf=&sa=0&dbf=0)  2016 | Cross-Sectional Study | ★★★☆☆ | ★☆ | ★★☆ | 6/10 |
| Paker [(15)](https://sciwheel.com/work/citation?ids=16590955&pre=&suf=&sa=0&dbf=0)  2018 | Cross-Sectional Study | ★★★☆☆ | ★★ | ★★☆ | 7/10 |
| Rosenberg [(16)](https://sciwheel.com/work/citation?ids=1499793&pre=&suf=&sa=0&dbf=0)  2016 | Cross-Sectional Study | ★★★★☆ | ★★ | ★★☆ | 8/10 |
| Scharre [(17)](https://sciwheel.com/work/citation?ids=3296761&pre=&suf=&sa=0&dbf=0)  2016 | Cross-Sectional Study | ★★★☆☆ | ★★ | ★★☆ | 7/10 |
| Schnittger [(18)](https://sciwheel.com/work/citation?ids=8574132&pre=&suf=&sa=0&dbf=0)  2012 | Cross-Sectional Study | ★★★★☆ | ★☆ | ★★☆ | 7/10 |
| Schrag [(19)](https://sciwheel.com/work/citation?ids=2407123&pre=&suf=&sa=0&dbf=0)  2015 | Case-Control Study | ★★★★ | ★★ | ★★★ | 9/9 |
| Si [(20)](https://sciwheel.com/work/citation?ids=8575013&pre=&suf=&sa=0&dbf=0)  2019 | Cross-Sectional Study | ★★★★☆ | ★★ | ★★☆ | 8/10 |
| Soysall [(21)](https://sciwheel.com/work/citation?ids=14299967&pre=&suf=&sa=0&dbf=0)  2023 | Cross-Sectional Study | ★★★★☆ | ★☆ | ★★☆ | 7/10 |
| Swiatkowska [(22)](https://sciwheel.com/work/citation?ids=9689507&pre=&suf=&sa=0&dbf=0)  2022 | Case-Control Study | ★★★★ | ★☆ | ★★★ | 8/9 |
| Wang [(23)](https://sciwheel.com/work/citation?ids=13263451&pre=&suf=&sa=0&dbf=0)  2022 | Cross-Sectional Study | ★★★★☆ | ★★ | ★★☆ | 8/10 |
| Yamada [(24)](https://sciwheel.com/work/citation?ids=8574373&pre=&suf=&sa=0&dbf=0)  2014 | Cross-Sectional Study | ★★★★☆ | ★★ | ★★☆ | 8/10 |
| Zahirovic [(25)](https://sciwheel.com/work/citation?ids=16590981&pre=&suf=&sa=0&dbf=0)  2019 | Prospective Cohort Study | ★★★★ | ★☆ | ★★★ | 8/9 |
| Zhang [(26)](https://sciwheel.com/work/citation?ids=16590949&pre=&suf=&sa=0&dbf=0)  2023 | Cross-Sectional Study | ★★★☆☆ | ★★ | ★★☆ | 7/10 |


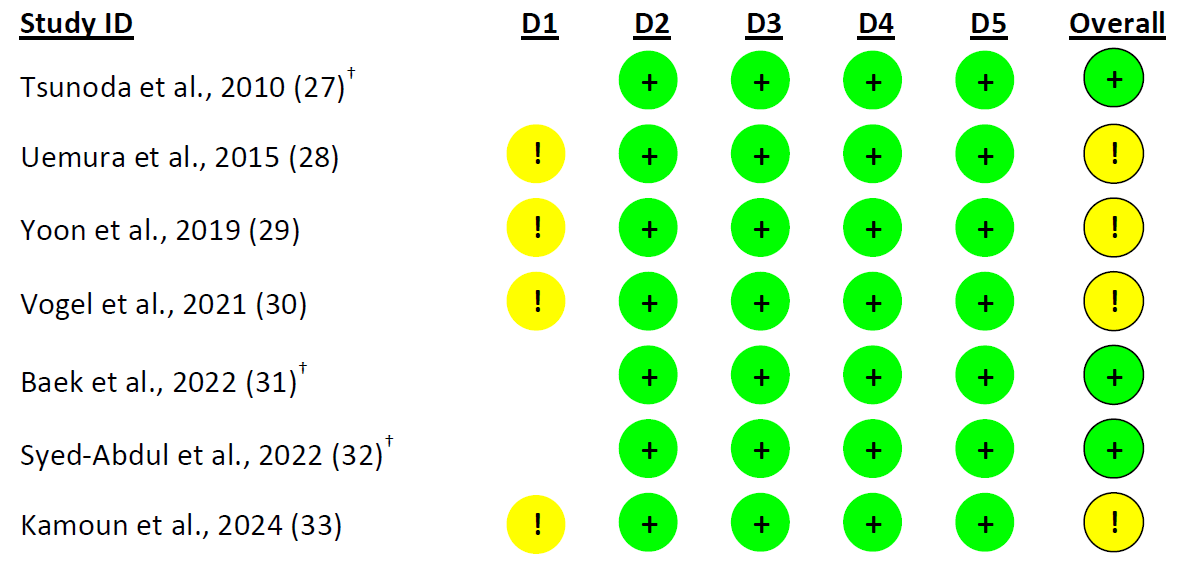


Figure A1. Risk of bias in the included experimental studies (n=7)

+: Low risk; !: Some concerns; ^†^: Quasi-experimental studies (one group pretest–posttest) were assessed without D1; D1: Randomization process; D2: Deviations from the intended interventions; D3: Missing outcome data; D4: Measurement of the outcome; D5: Selection of the reported result

References

[1.    Alasmari RS, Hassani HA, Almalky NA, Bokhari AF, Al Zahrani A, Hafez AA. Risk factors for fall among the elderly with diabetes mellitus type 2 in Jeddah, Saudi Arabia, 2022: a cross-sectional study. Ann Med Surg (Lond). 2023 Mar 9;85(3):412–7.](https://sciwheel.com/work/bibliography/16606276)

[2.    Amjad H, Snyder SH, Wolff JL, Oh E, Samus QM. Before hospice: symptom burden, dementia, and social participation in the last year of life. J Palliat Med. 2019 Sep;22(9):1106–14.](https://sciwheel.com/work/bibliography/8574923)

[3.    Arena JE, Weigand SD, Whitwell JL, Hassan A, Eggers SD, Höglinger GU, et al. Progressive supranuclear palsy: progression and survival. J Neurol. 2016 Feb;263(2):380–9.](https://sciwheel.com/work/bibliography/2984774)

[4.    Catikkas NM, Tunc M, Soysal P. The prevalence of excessive daytime sleepiness and associated factors in older diabetic patients. Aging Clin Exp Res. 2023 Dec 8;35(12):3205–14.](https://sciwheel.com/work/bibliography/16590956)

[5.    Chen P-H, Cheng F-Y, Cheng S-J, Shaw J-S. Predicting Cognitive Decline in Parkinson’s Disease with Mild Cognitive Impairment: A One-Year Observational Study. Parkinsons Dis. 2020 Oct 28;2020:8983960.](https://sciwheel.com/work/bibliography/16590948)

[6.    Dokuzlar O, Soysal P, Isik AT. Association between serum vitamin B12 level and frailty in older adults. North Clin Istanb. 2017 May 10;4(1):22–8.](https://sciwheel.com/work/bibliography/7731911)

[7.    Engin S, Ozturk M, Engin N, Kulaksizoglu IB. Dark side of the town: depressive symptoms in disadvantaged senior citizens. J Nutr Health Aging. 2010 Jun;14(6):483–7.](https://sciwheel.com/work/bibliography/8576076)

[8.    Ganidagli S, Ozturk E, Ozturk ZA. Risk factors of poor sleep quality in older adults: an analysis based on comprehensive geriatric assessment. Curr Med Res Opin. 2023 May;39(5):701–6.](https://sciwheel.com/work/bibliography/15671536)

[9.    Gazzola JM, Aratani MC, Doná F, Macedo C, Fukujima MM, Ganança MM, et al. Factors relating to depressive symptoms among elderly people with chronic vestibular dysfunction. Arq Neuropsiquiatr. 2009 Jun;67(2B):416–22.](https://sciwheel.com/work/bibliography/12411209)

[10.   Lichter DG, Benedict RHB, Hershey LA. Freezing of gait in parkinson’s disease: risk factors, their interactions, and associated nonmotor symptoms. Parkinsons Dis. 2021 Jan 12;2021:8857204.](https://sciwheel.com/work/bibliography/14104016)

[11.   Louis ED, Wise A, Alcalay RN, Rao AK, Factor-Litvak P. Essential tremor-Parkinson’s disease: A double whammy. J Neurol Sci. 2016 Jul 15;366:47–51.](https://sciwheel.com/work/bibliography/16590942)

[12.   Nisser J, Derlien S, Bublak P, Schwab M, Witte OW, Kesper K, et al. Systematic quantitative assessment of motor function in clinically isolated REM sleep behaviour disorder: A diagnostic window into early alpha-synucleinopathies. J Sleep Res. 2022 Apr;31(2):e13459.](https://sciwheel.com/work/bibliography/16590959)

[13.   Güner Oytun M, Topuz S, Baş AO, Çöteli S, Kahyaoğlu Z, Boğa İ, et al. Relationships of Fall Risk With Frailty, Sarcopenia, and Balance Disturbances in Mild-to-Moderate Alzheimer’s Disease. J Clin Neurol. 2023 May;19(3):251–9.](https://sciwheel.com/work/bibliography/16606280)

[14.   Pahwa AK, Andy UU, Newman DK, Stambakio H, Schmitz KH, Arya LA. Noctural Enuresis as a Risk Factor for Falls in Older Community Dwelling Women with Urinary Incontinence. J Urol. 2016 May;195(5):1512–6.](https://sciwheel.com/work/bibliography/8573920)

[15.   PAKER N, İLKE ŞEN E, BUĞDAYCI D, FERHATOSMANOĞLU A. PREDICTORS OF FUNCTIONAL BALANCE IN OLDER ADULTS LIVING IN A COMMUNITY. Turkish Journal of Geriatrics / Türk Geriatri Dergisi; 2018.](https://sciwheel.com/work/bibliography/16590955)

[16.   Rosenberg DE, Bellettiere J, Gardiner PA, Villarreal VN, Crist K, Kerr J. Independent associations between sedentary behaviors and mental, cognitive, physical, and functional health among older adults in retirement communities. J Gerontol A Biol Sci Med Sci. 2016 Jan;71(1):78–83.](https://sciwheel.com/work/bibliography/1499793)

[17.   Scharre DW, Chang S-I, Nagaraja HN, Park A, Adeli A, Agrawal P, et al. Paired Studies Comparing Clinical Profiles of Lewy Body Dementia with Alzheimer’s and Parkinson’s Diseases. J Alzheimers Dis. 2016 Oct 4;54(3):995–1004.](https://sciwheel.com/work/bibliography/3296761)

[18.   Schnittger RIB, Walsh CD, Casey A-M, Wherton JP, McHugh JE, Lawlor BA. Psychological distress as a key component of psychosocial functioning in community-dwelling older people. Aging Ment Health. 2012;16(2):199–207.](https://sciwheel.com/work/bibliography/8574132)

[19.   Schrag A, Horsfall L, Walters K, Noyce A, Petersen I. Prediagnostic presentations of Parkinson’s disease in primary care: a case-control study. Lancet Neurol. 2015 Jan;14(1):57–64.](https://sciwheel.com/work/bibliography/2407123)

[20.   Si H, Wang C, Jin Y, Tian X, Qiao X, Liu N, et al. Prevalence, Factors, and Health Impacts of Chronic Pain Among Community-Dwelling Older Adults in China. Pain Manag Nurs. 2019 Aug;20(4):365–72.](https://sciwheel.com/work/bibliography/8575013)

[21.   Soysal P, Koc Okudur S, Uslu F, Smith L. Functional loss and worsening geriatric assessment parameters are more common in dementia with Lewy bodies than Alzheimer’s disease. Psychogeriatrics. 2023 Jan;23(1):77–85.](https://sciwheel.com/work/bibliography/14299967)

[22.   Swiatkowska I, Henckel J, Sabah SA, Hart AJ. Self-Reported Neurotoxic Symptoms in Hip Arthroplasty Patients With Highly Elevated Blood Cobalt: A Case-Control Study. J Patient Saf. 2022 Jan 1;18(1):e10–7.](https://sciwheel.com/work/bibliography/9689507)

[23.   Wang QX, Ye ZM, Wu WJ, Zhang Y, Wang CL, Zheng HG. Association of Fear of Falling With Cognition and Physical Function in Community-Dwelling Older Adults. Nurs Res. 2022 Oct 1;71(5):387–93.](https://sciwheel.com/work/bibliography/13263451)

[24.   Yamada Y, Vlachova M, Richter T, Finne-Soveri H, Gindin J, van der Roest H, et al. Prevalence and correlates of hearing and visual impairments in European nursing homes: results from the SHELTER study. J Am Med Dir Assoc. 2014 Oct;15(10):738–43.](https://sciwheel.com/work/bibliography/8574373)

[25.   Zahirovic I, Torisson G, Wattmo C, Londos E. Survival among the older adults with clinical signs of Lewy body dementia in 40 Swedish nursing homes: a 6-year follow-up study. BMJ Open. 2019 May 30;9(5):e028010.](https://sciwheel.com/work/bibliography/16590981)

[26.   Zhang Y, Ye M, Wang X, Wu J, Wang L, Zheng G. Age differences in factors affecting fear of falling among community-dwelling older adults: A cross-sectional study. Geriatr Nurs. 2023;49:74–80.](https://sciwheel.com/work/bibliography/16590949)

[27.   Uemura SI, Kanbayashi T, Wakasa M, Satake M, Ito W, Shimizu K, et al. Residual effects of zolpidem, triazolam, rilmazafone and placebo in healthy elderly subjects: a randomized double-blind study. Sleep Med. 2015 Nov;16(11):1395–402.](https://sciwheel.com/work/bibliography/16606288)

[28.   Yoon J-R, Ha G-C, Kang S-J, Ko K-J. Effects of 12-week resistance exercise and interval training on the skeletal muscle area, physical fitness, and mental health in old women. J Exerc Rehabil. 2019 Dec 31;15(6):839–47.](https://sciwheel.com/work/bibliography/16590947)

[29.   Vogel O, Niederer D, Vogt L. Multimodal exercise effects in older adults depend on sleep, movement biography, and habitual physical activity: A randomized controlled trial. Front Aging Neurosci. 2021 Oct 22;13:722799.](https://sciwheel.com/work/bibliography/13170619)

[30.   Kamoun A, Yahia A, Farjallah MA, Maaloul R, Marzougui H, Bouaziz M, et al. Concurrent training associated with moderate walnut consumption improved isokinetic strength, subjective sleep quality, cognitive performance and postural balance in elderly active men: a randomized controlled trial. Aging Clin Exp Res. 2024 Feb 29;36(1):50.](https://sciwheel.com/work/bibliography/16590940)

[31.   Tsunoda K, Uchida H, Suzuki T, Watanabe K, Yamashima T, Kashima H. Effects of discontinuing benzodiazepine-derivative hypnotics on postural sway and cognitive functions in the elderly. Int J Geriatr Psychiatry. 2010 Dec;25(12):1259–65.](https://sciwheel.com/work/bibliography/2357295)

[32.   Baek J-E, Jung J-H, Shin H-J, Kim S-H, Sung S-Y, Park S-J, et al. Effects of Forest Healing Anti-Aging Program on Psychological, Physiological, and Physical Health of Older People with Mild Cognitive Impairment. Int J Environ Res Public Health. 2022 Apr 16;19(8).](https://sciwheel.com/work/bibliography/16590939)

[33.   Syed-Abdul MM, McClellan CL, Parks EJ, Ball SD. Effects of a resistance training community programme in older adults. Ageing Soc. 2022 Aug;42(8):1863–78.](https://sciwheel.com/work/bibliography/16606292)

[34.   Spira AP, Beaudreau SA, Stone KL, Kezirian EJ, Lui L-Y, Redline S, et al. Reliability and validity of the Pittsburgh Sleep Quality Index and the Epworth Sleepiness Scale in older men. J Gerontol A Biol Sci Med Sci. 2012 Apr;67(4):433–9.](https://sciwheel.com/work/bibliography/2705811)

[35.   Herman T, Giladi N, Hausdorff JM. Properties of the “timed up and go” test: more than meets the eye. Gerontology. 2011;57(3):203–10.](https://sciwheel.com/work/bibliography/2156155)

[36.   Rockwood K, Awalt E, Carver D, MacKnight C. Feasibility and measurement properties of the functional reach and the timed up and go tests in the Canadian study of health and aging. J Gerontol A Biol Sci Med Sci. 2000 Feb;55(2):M70-3.](https://sciwheel.com/work/bibliography/5929751)

[37.   Shumway-Cook A, Brauer S, Woollacott M. Predicting the probability for falls in community-dwelling older adults using the Timed Up & Go Test. Phys Ther. 2000 Sep;80(9):896–903.](https://sciwheel.com/work/bibliography/2043839)

[38.   Berg K. Measuring balance in the elderly: preliminary development of an instrument. Physiotherapy Canada. 1989 Nov;41(6):304–11.](https://sciwheel.com/work/bibliography/5392304)

[39.   Park J, Koh S-B, Kim HJ, Oh E, Kim J-S, Yun JY, et al. Validity and reliability study of the korean tinetti mobility test for parkinson’s disease. J Mov Disord. 2018 Jan 23;11(1):24–9.](https://sciwheel.com/work/bibliography/11294273)

[40.   Canbek J, Fulk G, Nof L, Echternach J. Test-retest reliability and construct validity of the tinetti performance-oriented mobility assessment in people with stroke. J Neurol Phys Ther. 2013 Mar;37(1):14–9.](https://sciwheel.com/work/bibliography/5373815)

[41.   Sterke CS, Huisman SL, van Beeck EF, Looman CWN, van der Cammen TJM. Is the Tinetti Performance Oriented Mobility Assessment (POMA) a feasible and valid predictor of short-term fall risk in nursing home residents with dementia? Int Psychogeriatr. 2010 Mar;22(2):254–63.](https://sciwheel.com/work/bibliography/5753811)

[42.   Powell LE, Myers AM. The Activities-specific Balance Confidence (ABC) Scale. J Gerontol A Biol Sci Med Sci. 1995 Jan;50A(1):M28-34.](https://sciwheel.com/work/bibliography/2097247)

[43.   McDowell I, Kristjansson B, Hill GB, Hébert R. Community screening for dementia: the Mini Mental State Exam (MMSE) and Modified Mini-Mental State Exam (3MS) compared. J Clin Epidemiol. 1997 Apr;50(4):377–83.](https://sciwheel.com/work/bibliography/10112654)

[44.   Hopp GA, Dixon RA, Grut M, Bäckman L. Longitudinal and psychometric profiles of two cognitive status tests in very old adults. Journal of Clinical Psychology. 1997 Nov 1;](https://sciwheel.com/work/bibliography/17921165)

[45.   Seo EH, Lee DY, Lee JH, Choo IH, Kim JW, Kim SG, et al. Total scores of the CERAD neuropsychological assessment battery: validation for mild cognitive impairment and dementia patients with diverse etiologies. Am J Geriatr Psychiatry. 2010 Sep;18(9):801–9.](https://sciwheel.com/work/bibliography/2405022)

[46.   Abdolahi A, Scoglio N, Killoran A, Dorsey ER, Biglan KM. Potential reliability and validity of a modified version of the Unified Parkinson’s Disease Rating Scale that could be administered remotely. Parkinsonism Relat Disord. 2013 Feb;19(2):218–21.](https://sciwheel.com/work/bibliography/8915200)

[47.   Scharre DW, Chang S-I, Murden RA, Lamb J, Beversdorf DQ, Kataki M, et al. Self-administered Gerocognitive Examination (SAGE): a brief cognitive assessment Instrument for mild cognitive impairment (MCI) and early dementia. Alzheimer Dis Assoc Disord. 2010;24(1):64–71.](https://sciwheel.com/work/bibliography/5845782)

[48.   Nasreddine ZS, Phillips NA, Bédirian V, Charbonneau S, Whitehead V, Collin I, et al. The Montreal Cognitive Assessment, MoCA: A brief screening tool for mild cognitive impairment. J Am Geriatr Soc. 2005 Apr;53(4):695–9.](https://sciwheel.com/work/bibliography/1028118)

[49.   Ferreira S, Raimundo A, Marmeleira J. Test-retest reliability of the functional reach test and the hand grip strength test in older adults using nursing home services. Ir J Med Sci. 2021 Nov;190(4):1625–32.](https://sciwheel.com/work/bibliography/15171828)

[50.   Muñoz-Mendoza CL, Cabañero-Martínez MJ, Millán-Calenti JC, Cabrero-García J, López-Sánchez R, Maseda-Rodríguez A. Reliability of 4-m and 6-m walking speed tests in elderly people with cognitive impairment. Arch Gerontol Geriatr. 2011;52(2):e67-70.](https://sciwheel.com/work/bibliography/14897564)
